# Cell Surface Area and Membrane Folding in Glioblastoma Cell Lines Differing in *PTEN* and *p53* Status

**DOI:** 10.1371/journal.pone.0087052

**Published:** 2014-01-31

**Authors:** Simon Memmel, Vladimir L. Sukhorukov, Marcus Höring, Katherine Westerling, Vanessa Fiedler, Astrid Katzer, Georg Krohne, Michael Flentje, Cholpon S. Djuzenova

**Affiliations:** 1 Lehrstuhl für Biotechnologie und Biophysik, Universität Würzburg, Am Hubland, Würzburg, Germany; 2 Department of Radiation Oncology, University Hospital Würzburg, Würzburg, Germany; 3 Elektronenmikroskopie, Biozentrum, Universität Würzburg, Am Hubland, Würzburg, Germany; University of Portsmouth, School of Pharmacy & Biomedical Sciences, United Kingdom

## Abstract

Glioblastoma multiforme (GBM) is characterized by rapid growth, invasion and resistance to chemo−/radiotherapy. The complex cell surface morphology with abundant membrane folds, microvilli, filopodia and other membrane extensions is believed to contribute to the highly invasive behavior and therapy resistance of GBM cells. The present study addresses the mechanisms leading to the excessive cell membrane area in five GBM lines differing in mutational status for PTEN and p53. In addition to scanning electron microscopy (SEM), the membrane area and folding were quantified by dielectric measurements of membrane capacitance using the single-cell electrorotation (ROT) technique. The osmotic stability and volume regulation of GBM cells were analyzed by video microscopy. The expression of PTEN, p53, mTOR and several other marker proteins involved in cell growth and membrane synthesis were examined by Western blotting. The combined SEM, ROT and osmotic data provided independent lines of evidence for a large variability in membrane area and folding among tested GBM lines. Thus, DK-MG cells (wild type p53 and wild type PTEN) exhibited the lowest degree of membrane folding, probed by the area-specific capacitance *C*_m_ = 1.9 µF/cm^2^. In contrast, cell lines carrying mutations in both p53 and PTEN (U373-MG and SNB19) showed the highest *C*_m_ values of 3.7–4.0 µF/cm^2^, which corroborate well with their heavily villated cell surface revealed by SEM. Since PTEN and p53 are well-known inhibitors of mTOR, the increased membrane area/folding in mutant GBM lines may be related to the enhanced protein and lipid synthesis due to a deregulation of the mTOR-dependent downstream signaling pathway. Given that membrane folds and extensions are implicated in tumor cell motility and metastasis, the dielectric approach presented here provides a rapid and simple tool for screening the biophysical cell properties in studies on targeting chemo- or radiotherapeutically the migration and invasion of GBM and other tumor types.

## Introduction

Glioblastoma multiforme (GBM) is the most common and aggressive human brain cancer, accounting for about 15% of all intracranial tumors [1]–[3]. Despite advances in chemotherapy, surgical and radiation oncology, the prognosis of this tumor remains very poor [4]–[6]. The unfavorable clinical outcome is largely attributed to the highly invasive nature of GBM cells [7], based on their remarkable ability to invade healthy brain tissue and migrate extensively within the CNS [8], thus escaping surgical removal as well as exposure to radiation and chemotherapy [6], [9].

Numerous light and electron microscopic studies reveal complex surface topography of primary glioma cells and GBM cell lines, which exhibit dense microvilli, membrane folds, filopodia and other membrane protrusions [10]–[13]. According to Friedl & Wolf (2003), these membrane extensions may be implicated in many tumor cell activities, including adhesion, spreading, migration and invasion [14]. Moreover, microvilli have been reported to protect GBM cells from being killed by cytolytic effector cells of the immune system [15], [16].

It is evident that membrane folds and microvilli along with a typically flattened shape of GBM cells [17], [18] create a significant excess in the cell surface area as compared to a smooth spherical cell of similar volume. Consistent with microscopic data, recent dielectric studies also reveal a much greater membrane folding in glioblastoma and other cancer cells, as compared to normal blood cells [19], [20]. Among other reasons, the excessive cell membrane area may be a result of an increased *de novo* lipogenesis typical for malignant cells [21]–[23].

Although derived from the same tumor type, glioblastoma cell lines exhibit a wide range of morphological diversity, including fibroblastic, epithelial, glial and other patterns [24]–[26]. The morphological cell properties and membrane folding reflect most likely the individual genotypes of the tumors of origin and can therefore have predictive value for malignant behavior. Common genetic alterations in glioblastoma include both amplification of oncogenes (e.g. *EGFR, CDK4*) and deletion of tumor suppressor genes, most frequently *PTEN* and *TP53* [27]–[30]. Moreover, the increased aggressiveness of tumors deficient in both *PTEN* and *p53* suggests that their combined loss may result in an increased tumorigenic potential [31].

Until now, molecular pathogenesis studies of GBM cells revealed no apparent correlations between morphological and genetic data [24], [32]–[35]. Particularly, the mechanisms responsible for the excessive membrane folding and microvilli expression in GBM cells remain unclear. To address this issue, we explore in the present study the plasma membrane morphology in five GBM lines differing in the mutational status of *PTEN* and *p53*. In addition to scanning electron microscopy (SEM), we applied the electrorotation (ROT) technique to quantify the area-specific membrane capacity *C*_m_ [µF/cm^2^], related to membrane folding, and the whole-cell capacitance *C*_C_ [pF], which reflects the total membrane area [36]–[38]. The observed large differences in both *C*_m_ and *C*_C_ among GBM lines, revealed by ROT, prompted us to examine in detail the possible signaling pathways involved in the regulation of the cell line-specific membrane morphology.

## Materials and Methods

### Cell Culture

The set of 5 human glioblastoma (GBM) cell lines studied here includes DK-MG, GaMG, U87-MG, U373-MG, and SNB19 cells. All cell lines were obtained from ATCC and routinely cultured under standard conditions (5% CO_2_, 37°C) in Complete Growth Medium (CGM), which was either Dulbecco’s modified Eagle’s medium (DK-MG, GaMG, SNB19) or minimum essential medium (U87-MG, U373-MG), supplemented with 10% FBS. Mutations of the tumor suppressors PTEN and p53 in the tested cell lines are summarized in Table 1 given in the Results section.

**Table 1:** Mutational status,* cell radius and dielectric properties of 5 GBM lines studied here.

Cell line	p53	PTEN	*Radius* ± SE µm	*C*_m_ ± SE µF/cm^2^	*C*_C_ ± SE pF	Folding φ	*N*, cell number
**DK-MG**	wild type	wild type	6.3±0.1	1.88±0.07	9.4±0.2	2.38	420
**GaMG**	**mutated**	wild type	8.5±0.1	3.17±0.12	28.8±0.9	4.00	300
**U87-MG**	wild type	**mutated**	6.7±0.1	2.82±0.09	15.9±0.3	3.50	420
**U373-MG**	**mutated**	**mutated**	7.1±0.1	4.00±0.12	25.3±0.7	5.25	360
**SNB19**	**mutated**	**mutated**	6.8±0.1	3.71±0.15	21.6±0.7	4.63	360

* The mutation data for p53 and PTEN genes were acquired from COSMIC database (Catalogue of Somatic Mutations in Cancer).

Cells were passaged thrice weekly by seeding at about 10^4^ cells per cm^2^ and grown to subconfluence before being harvested with trypsin-EDTA detachment. After washing with CGM, the cells were spun down and resuspended in either inositol-substituted medium for electrorotation or in phosphate buffer saline for electrosizing. Cell morphology was evaluated in monolayers by phase contrast microscopy, and in suspension by electrosizing using a Coulter-like CASY counter (Schärfe System, Reutlingen, Germany).

### Scanning Electron Microscopy (SEM)

**Figure 1:**
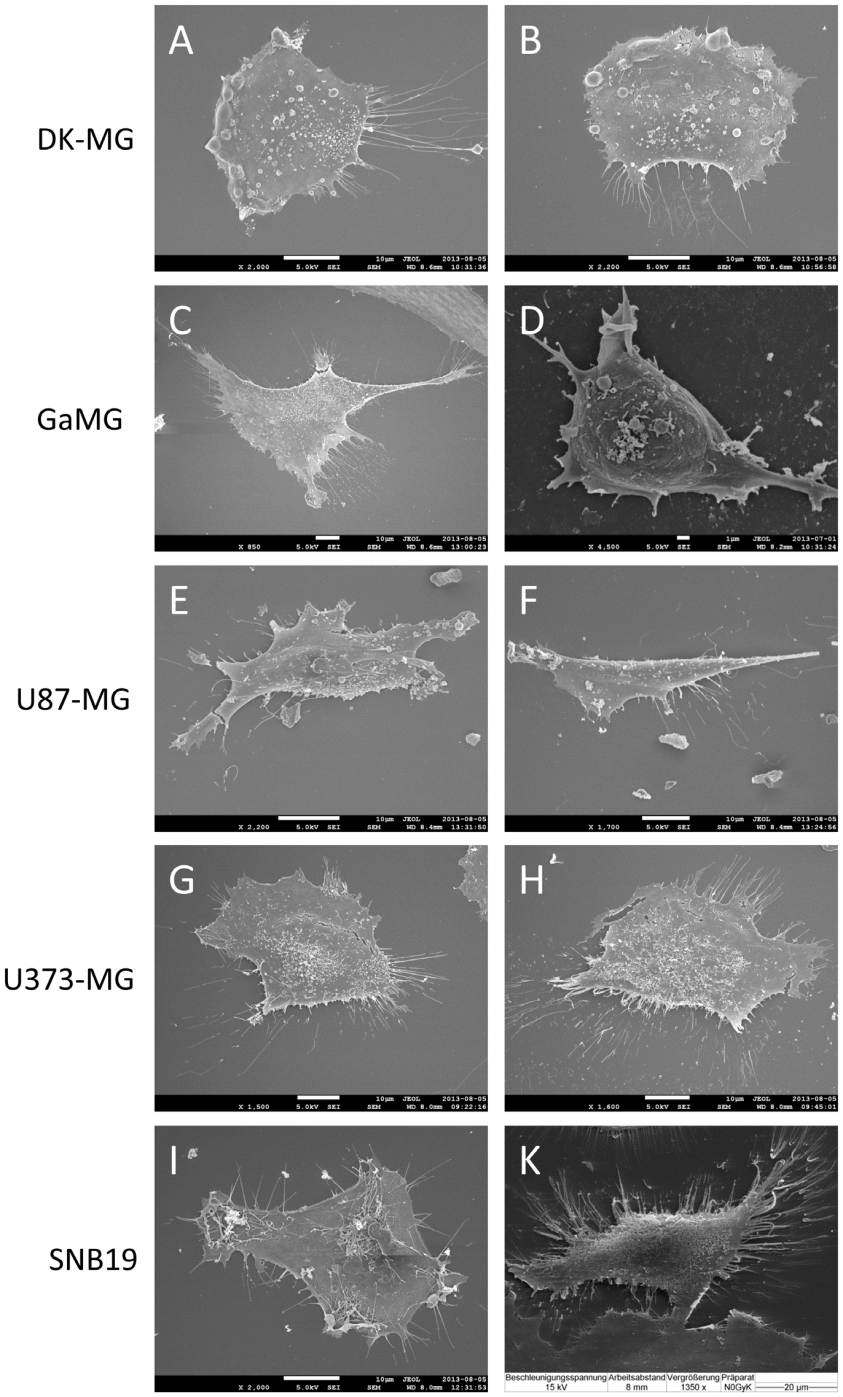
Representative scanning electron micrographs of 5 GBM lines used in the present study. For each cell line 2 views are shown, with the scale bars given in the bottom edges of each image. Scale bars in A–C and E–I correspond to 10 µm, in D and K the scale bars are 1 and 20 µm, respectively.

For SEM, cells were seeded on microscope cover glass and fixed by addition of 6.25% glutaraldehyde in 50 mM phosphate buffer (pH 7.2) for 10 min at RT and subsequently at 4°C overnight [39]. After a washing step, samples were dehydrated stepwise in acetone, critical point dried and sputtered with gold/palladium before SEM analysis (JEOL JSM 7500F). Although critical point drying is a gentle method, shrinkage within cells grown on cover slips cannot always been prevented during the drying process. Shrinkage results in the formation of local cracks in the cells (see Fig. 1C, E, G, H).

### Electrorotation and Derivation of Membrane Parameters

Electrorotation (ROT) spectra were measured in a microstructured four electrode chamber, described in detail earlier [37]. The microchamber is arranged as a planar array of circular plane electrodes spaced by 300 µm. The electrodes were driven by four 90° phase-shifted, rectangular signals from a pulse generator (Hewlett-Packard, Boeblingen, Germany) with 2.5–4.8 V_PP_ amplitude over the frequency range from 100 Hz to 150 MHz. A drop of cell suspension (50–70 µL) was added to the ROT chamber, and a coverslip was placed gently over its center. The cell rotation was observed using a BX50 Olympus microscope (Hamburg, Germany). ROT spectra were monitored by decreasing the field frequency in steps (five frequency points per decade). At each field frequency, the rotation speed of lone cells located near the center of the chamber was determined using a stopwatch. The ROT spectra, i.e., frequency dependencies of the ROT speed Ω [radian/s], were normalized to the field strength of 100 V/cm.

Measurements of the field frequency *f*_c1_ that induced fastest anti-field cell rotation were performed by the contra-rotating fields (CRF) technique [40]. In contrast to the regular ROT, the cell response to CRF is proportional to the differential of the ROT spectrum (i.e. ∂Ω/∂*f*). The CRF method permits accurate and rapid determination of the maximum rotation frequency *f*_c1_, at which ∂Ω/∂*f* = 0 and the cell stop rotating. In the CRF experiments, 10 µL of cell suspension was added to a four-electrode chamber described previously [36]. The electrode spacing was ∼1.2 mm. The chamber is open at the top for rapid sample replacement. The cells were viewed with an inverted Leitz-Labovert microscope through a 100× oil-immersion objective. Cell radii were determined with a calibrated eyepiece micrometer. Conductivity within the chamber was monitored by a conductometer connected to two opposite electrodes.

The ROT spectra of living cells can be presented as a superposition of several Lorentzian curves caused by the Maxwell-Wagner dispersions at the various dielectric interfaces, e.g. at the plasma and nuclear membranes [41], [42]:
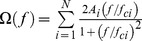
(1)where *N*, *A*_i_ and *f*_ci_ are the number, magnitudes and characteristic frequencies of the ROT peaks, respectively. Providing that the Maxwell-Wagner relaxation processes are widely separated in the frequency domain, the ROT spectrum of human and other mammalian can exhibit 2–3 peaks in the kHz-MHz range.

The theory of single cell electrorotation gives the following relationship between the characteristic frequency of anti-field electrorotation *f*_c1_, the cell radius *a*, the area-specific membrane capacity *C*_m_ [µF/cm^2^] and conductance *G*_m_ [mS/cm^2^] [37]:
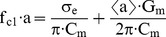
(2)where <*a*> is the mean cell radius, *σ*_e_ [µS/cm] is the external conductivity of suspending medium, which is assumed to be held so low that *σ*_e_<<*σ*_i_ (*σ*_i_ is the intracellular conductivity). The membrane parameters *C*_m_ and *G*_m_ can be extracted by fitting Eq. 2 to the (*f*_c1_·*a*) data plotted against *σ*_e_.

Given that smooth plasma membrane has a capacitance of *C*_m0_ ≈ 0.8 µF/cm^2^, an effective folding factor can be introduced as *φ* = *C*_m_/*C*_0_ [19], [36], [43]. The folding factor *φ* describes the ratio of the actual cell membrane surface area to that of a smooth sphere of the same radius. From the *C*_m_ and radius data, the whole-cell capacitance *C*_C_ [pF] was also calculated as:
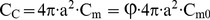
(3)


Unlike the *C*_m_ value, which represents the membrane capacitance per unit area, the parameter *C*_C_ accounts for the total electrically accessible cell membrane, including both smooth and folded membrane regions.

### Cell Volumetry

Hypotonic solutions used in the volumetric experiments contained either sucrose or sorbitol as the major osmolyte. The osmolality was adjusted to 50, 100 and 200 mosmol/kg (denoted hereafter as mOsm, using a cryoscopic osmometer (Gonotec, Berlin, Germany). Cell volume changes were measured by videomicroscopy using a flow chamber designed for rapid exchange of media [37]. Before measurements, an aliquot of cells suspended in isotonic CGM (∼300 mOsm) at a density of about 10^5^ cells/ml was injected into the chamber and the cells were allowed to settle and to adhere to the chamber floor for 10–15 min. The chamber was placed on the stage of a microscope (BX50, Olympus, Hamburg, Germany) and the cells were viewed with a 20× objective in transmitted light. The microscope was equipped with a CMOS video camera (UI-1410-C, UEye, Obersulm, Germany) connected to the video digitizing board of a personal computer. Images of cells were taken 1–2 min before and at various time intervals of 10 seconds up to 20 min after medium exchange. The cross-section areas of typically 9–10 cells per microscopic field were determined with an image analysis program Image J (Wayne Rasband, NIH, Maryland). At each time interval, the volume (*V*) of an individual cell was evaluated from its cross-section by assuming spherical geometry. The cell volume was normalized to the original isotonic volume (*V*_0_) as: ν = *V*/*V*_0_. The mean ν values (± SE) for a given experiment were calculated from a sequence of ∼160 images and plotted against time.

### Western Blot

For immunoblot analysis, whole cell lysates were prepared according standard procedures, 20–24 h after splitting the culture. Samples equivalent to 20****µg of protein were separated using 4–12% SDS-polyacrylamide pre-cast gels (Invitrogen, Karlsruhe, Germany) and transferred to nitrocellulose membranes according to manufacturer’s prescriptions. For protein detection, membranes were incubated with respective primary and species-specific peroxidase-labeled secondary antibodies according to standard protocols. The levels of protein expression were quantified using Image J program and normalized to the β-actin levels.

The primary antibodies used were: rabbit polyclonal anti-PTEN, rabbit polyclonal anti-PI3K p110, mouse monoclonal anti-phospho-AKT (Ser473), rabbit monoclonal anti-phospho-mTOR (Ser2448) (all from Cell Signaling, Danvers, MA), mouse monoclonal anti-p53 (Merck Chemicals Ltd., Nottingham, UK), mouse monoclonal anti-Fatty Acid Synthase (BD Biosciences, Heidelberg, Germany), mouse monoclonal anti-MDM2 (SMP14) (Santa Cruz Biotechnology, Inc., Heidelberg, Germany), mouse monoclonal anti-β-actin (Sigma, Deisenhofen, Germany). Secondary species-specific antibodies for Western blot were labelled with horseradish-peroxidase (DAKO, Hamburg, Germany).

## Results

### Scanning Electron Microscopy (SEM)

The SEM images of adherently growing GBM cells (Figure 1) reveal complex cell surface topography in all 5 cell lines. Depending on the cell line, the GBM cells exhibited various types of membrane protrusions, including microvilli, blebs and filopodia. As seen in the microphotographs, the formation of microvilli on the apical membrane varies greatly not only among cell lines, but also from cell to cell within a particular sample. Many U373-MG and SNB19 cells were found to possess dense microvilli distributed evenly over the entire cell surface (Figures 1H and 1K). In contrast, the plasma membrane of DK-MG cells displayed many bud-like surface protrusions (blebs) and irregular clusters of microvilli (Figures 1A and 1B), and appeared much smoother than U87-MG and SNB19 cells. In addition to their complex apical topography, all GBM cells exhibited a highly folded membrane at the lateral edges, such as in lamellipodia and filopodia, as well as long dendritic protrusions, most notably in U87-MG and SNB19 cells.

As shown previously [44], cell surface area and membrane folding can principally be estimated by counting individual microvilli on SEM images. In case of GBM cells, however, quantitative analysis of membrane area/folding from SEM images would be very cumbersome, if not impossible, because of the high microvilli expression, irregular cell shape and great morphological variability within and among the cell lines. Instead, we quantified the membrane area and folding by measuring, respectively, the whole-cell and area-specific membrane capacitance values using the electrorotation technique.

### Area-specific Membrane and Whole-cell Capacitance Probed by Electrorotation (ROT)

**Figure 2:**
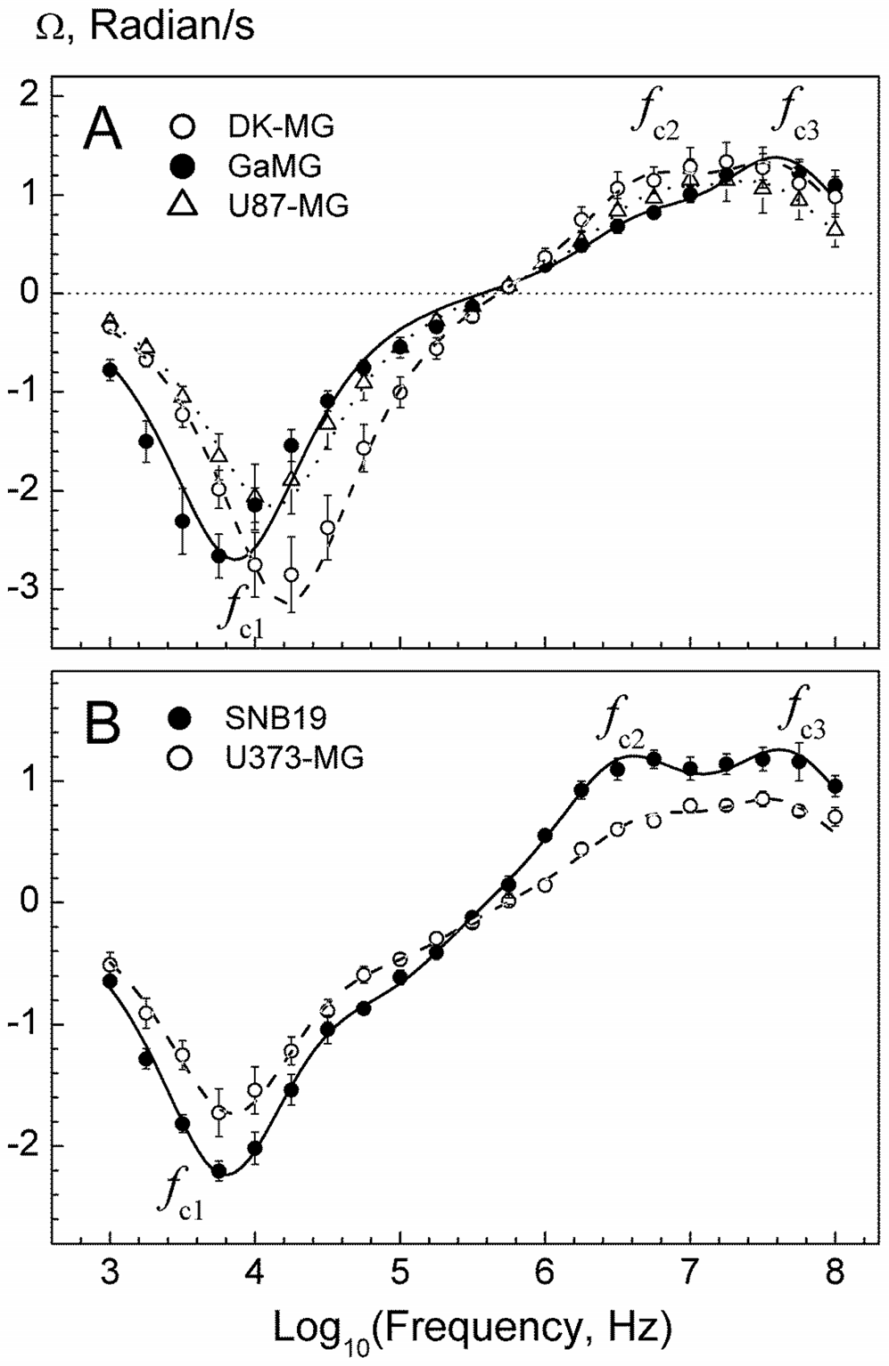
Rotation spectra of 5 GBM lines measured in isotonic 300-mOsm inositol solutions of the same conductivity of 50 µS/cm. Each spectrum (*symbols*) is the mean (±SE) of 8–10 single cell measurements. Curves are best fits with a double or triple Lorentzian function (Eq. 1). The fitted parameters are given in Table S1. The *f*_ci_ symbols denote three ROT peaks dominated by the plasma membrane charging (*f*_c1_), and by the polarization of the cytosol and cell nucleus (*f*_c2_ and *f*_c3_).

The following ROT experiments aimed to compare the area-specific plasma membrane capacitance *C*_m_ [µF/cm^2^] among 5 GBM lines by means of the contra-rotating field (CRF) technique. To this end, we first analyzed the complete ROT spectra of cells over a frequency range between 1 kHz and 100 MHz. As seen in Figure 2, all cell lines exhibited three ROT peaks, including an anti-field peak centered at the characteristic frequency *f*_c1_ of about 10 kHz and two co-field peaks (*f*_c2_ and *f*_c3_) in the MHz-range. A triple Lorentzian function (Eq. 1) fits very well the ROT spectra (*curves* in Figure 2), thus yielding the values for the peak frequencies (*f*_c1,2,3_) and magnitudes (*A*_1,2,3_), which are summarized in the Table S1.

Previous studies have shown that the low-frequency anti-field peak (*f*_c1_) is dominated by the capacitive charging of the plasma membrane, whereas the high-frequency co-field peaks (*f*_c2_ and *f*_c3_) arise from the polarization and/or dielectric dispersions in the cytoplasm and cell nucleus [36], [41]. As seen in Figure 2, the ROT spectra of GBM cells from 5 different cell lines display symmetrical anti-field peaks of Lorentzian shape. This result justifies the use of the CRF technique for the determination of the characteristic frequency of plasma membrane charging (*f*_c1_).

**Figure 3:**
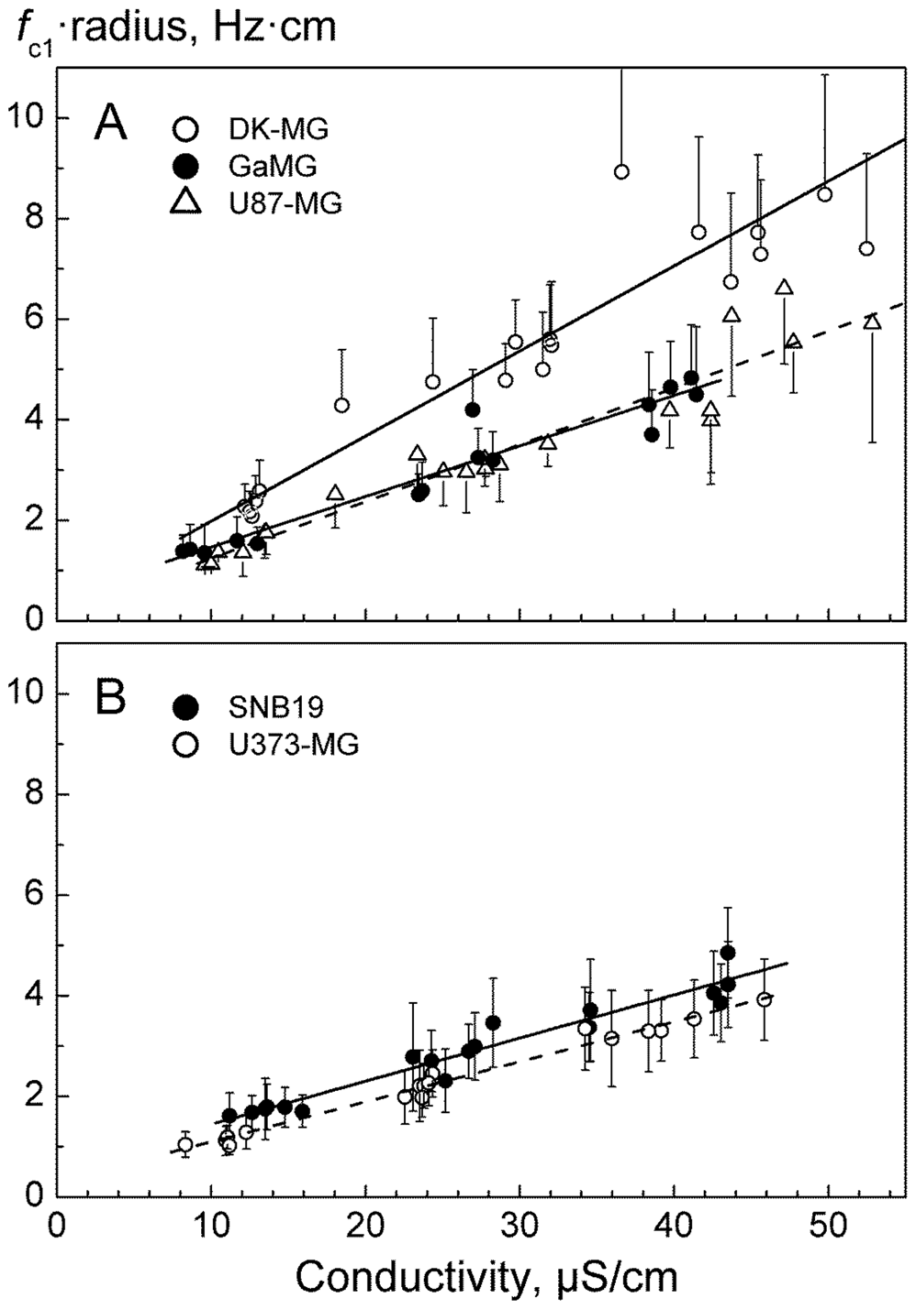
Cumulative plots of the radius-normalized *f*_c1_ values (*f*_c1_⋅a) of the indicated GBM lines *versus* the external conductivity σ_e_. The measurements were performed in isotonic 300-mOsm inositol medium. The *f*_c1_ data were obtained by the CRF-technique. Each symbol is the mean (±SE) from 20 cells measured at closely similar conductivities. The lines are best fits of Eq. 2 to the CRF data sets, each containing 300–420 cells. The line slopes are inversely proportional to *C*_m_ values. The steeper slope obtained for DK-MG cells implies a much smaller *C*_m_ = 1.9 µF/cm^2^ (*A*, *empty circles*), as compared to those of U373-MG (4.0 µF/cm^2^) and SNB19 cell lines (3.7 µF/cm^2^) (*B*). U87-MG and GaMG showed intermediate *C*_m_ values of 3.2 and 2.8 µF/cm^2^, respectively (*A*). The fitted *C*_m_ values are summarized in Table 1.

Using the CRF technique, we further analyzed the impact of medium conductivity σ_e_ on the plasma membrane peak (*f*_c1_). For each cell line, *f*_c1_-values and radii (*a*) of single cells were measured in large samples consisting of 300–420 cells. The cells were suspended in isotonic inositol medium (300 mOsm) of conductivity *σ*_e_ ranging between ∼10 and 50 µS/cm (Figure 3). Within this conductivity range, a linear relationship between the product *f*_c1_·*a* and σ_e_ is expected (Eq. 2) and is found in all cell lines (Figure 3). The *f*_c1_·*a* data of each cell line were fitted to Eq. 2 to calculate the mean area-specific membrane capacitance *C*_m_. The fitted *C*_m_ values for 5 GBM lines are summarized in Table 1.

Once the *C*_m_ and radius data were available, the whole cell capacitance *C*_C_ [pF] was calculated using Eq. 3. As suggested elsewhere [19], we also determined the plasma membrane folding factor φ, defined as the ratio φ = *C*_m_/*C*_m0_, where *C*_m0_ = 0.8 µF/cm^2^ is the capacitance of a smooth/flat membrane (*see* Discussion).

In isotonic medium, the 5 GBM lines exhibited very different *C*_m_ values ranging between 1.88 µF/cm^2^ in DK-MG and 4.0 µF/cm^2^ in U373-MG cells (Table 1). Exceeding by far the flat membrane capacitance of 0.8 µF/cm^2^, the large *C*_m_ values obtained here indicate the high degree of membrane folding *φ* in all GBM cells and also a large variation of this parameter among tested cell lines (2.38≤ *φ* ≤5.25). Particularly, the φ values larger than 3 obtained here for cell lines with mutant *PTEN* or *p53* status, or both, are clearly at the upper edge of the φ range measured in 60 tumor cell lines by dielectrophoresis [19].

For comparison, we also analyzed the plasma membrane folding in two non-malignant human cell lines, including the human embryonic kidney HEK293 line and the human fibroblast cell line HFIB-1 (both are adherently growing cell lines). As evident from the Fig. S3, the mean *C*_m_ values of 1.56±0.10 and 2.05±0.12 µF/cm^2^ obtained, respectively, for HEK293 and HFIB-1 cells, are similar to that of DK-MG cells (1.88 µF/cm^2^), i.e. the lowest *C*_m_ value among 5 tested GBM lines.

To verify our conclusion that the extremely high isotonic *C*_m_ values of GBM cells (1.9–4.0 µF/cm^2^), were really due to excessive membrane folding, we analyzed the impact of hypotonic cell swelling on *C*_m_. The rationale of these experiments was to prove whether, and to what extent, the hypotonic stretching and expected membrane unfolding would cause a reduction of *C*_m_ as commonly found in mammalian cells [36], [37].

In agreement with previous studies, our ROT experiments revealed a strong dependence of *C*_m_ on the external osmolality in all tested GBM lines (Figure S1 and Table S2). Thus, decreasing the osmolality from 300 to 50 mOsm caused the *C*_m_ of DK-MG cells to decrease from the isotonic *C*_m_ = 1.88 µF/cm^2^ to the hypotonic *C*_mh_ = 1.05 µF/cm^2^. In case of U373-MG cells, the same osmotic shift resulted in a much greater absolute and percentage reduction of *C*_m_ from 4.0 to 1.19 µF/cm^2^ (Tables 1 and S2). In general, the hypotonically stressed cells from different GBM lines showed similar *C*_mh_ values lying within a narrow range between 0.97 and 1.19 µF/cm^2^ (Table S2), even though the isotonic *C*_m_ data were widely different (1.88–4.00 µF/cm^2^, Table 1). Being somewhat larger than the flat-membrane *C*_m0_ = 0.8 µF/cm^2^, the hypotonic *C*_mh_ values of GBM cells suggest that, despite severe hypotonicity, the cell surface was not perfectly smooth. The remaining folds/microvilli can be explained by the large original membrane excess in GBM cells present under isotonic conditions.

Unlike *C*_m_, the whole-cell capacitance *C*_C_ of GBM cells did not show any significant changes despite considerable cell swelling in hypotonic medium, as evident from the comparison of isotonic cell radius and *C*_C_ values in Table 1 with the corresponding hypotonic data given in the Table S2. Given that *C*_C_ reflects the total membrane area, these findings indicate that the swelling-mediated increase in cell surface was achieved without incorporation of new material into the plasma membrane, but largely via membrane unfolding, which was detected by the marked *C*_m_ reduction mentioned above.

### Osmotic Properties of Glioblastoma Cells

The consistently low *C*_mh_ values obtained for all GBM lines in strongly hypotonic medium (Figure S1, Table S2) suggest that the microvilli and membrane folds, rather than other mechanisms, were responsible for the very high *isotonic C*_m_ values. These membrane extensions are well known to provide an instantaneous source of material to preserve the plasma membrane integrity during hypotonic swelling [36], [45]. Given the large excess of membrane area, all GBM cells used here may be expected to be capable of withstanding, without rupture, a harsh hypotonic stress.

To analyze the osmotic stability of GBM cells, we monitored by videomicroscopy their response to an acute hypotonic challenge over a wide tonicity range (50–300 mOsm), in inositol- or sucrose-substituted media. Inspection of the video recordings showed that in all tested cell lines the majority of cells withstood very well the imposed hypotonic stress. Even upon extreme swelling at the lowest osmolality of 50 mOsm (i.e. 6 times lower than physiological), the portions of hypotonically lysed cells were very low: 2–10% DK-MG, 0.5–5% U87-MG, 1–2% GaMG, 1–3% U373-MG, and ∼1% SNB19 cells. These data corroborate the results of ROT measurements (Figure S1) demonstrating that GBM cells possessed an electrically functioning plasma membrane under extreme swelling conditions in 50-mOsm medium. Milder hypotonic stress (100 mOsm) did not cause any notable cytolysis in all tested cell lines. The high osmotic stability of GBM cells clearly supports the presence of large plasma membrane reserves in folds/microvilli, revealed by SEM (Figure 1) and ROT (Table 1).

**Figure 4:**
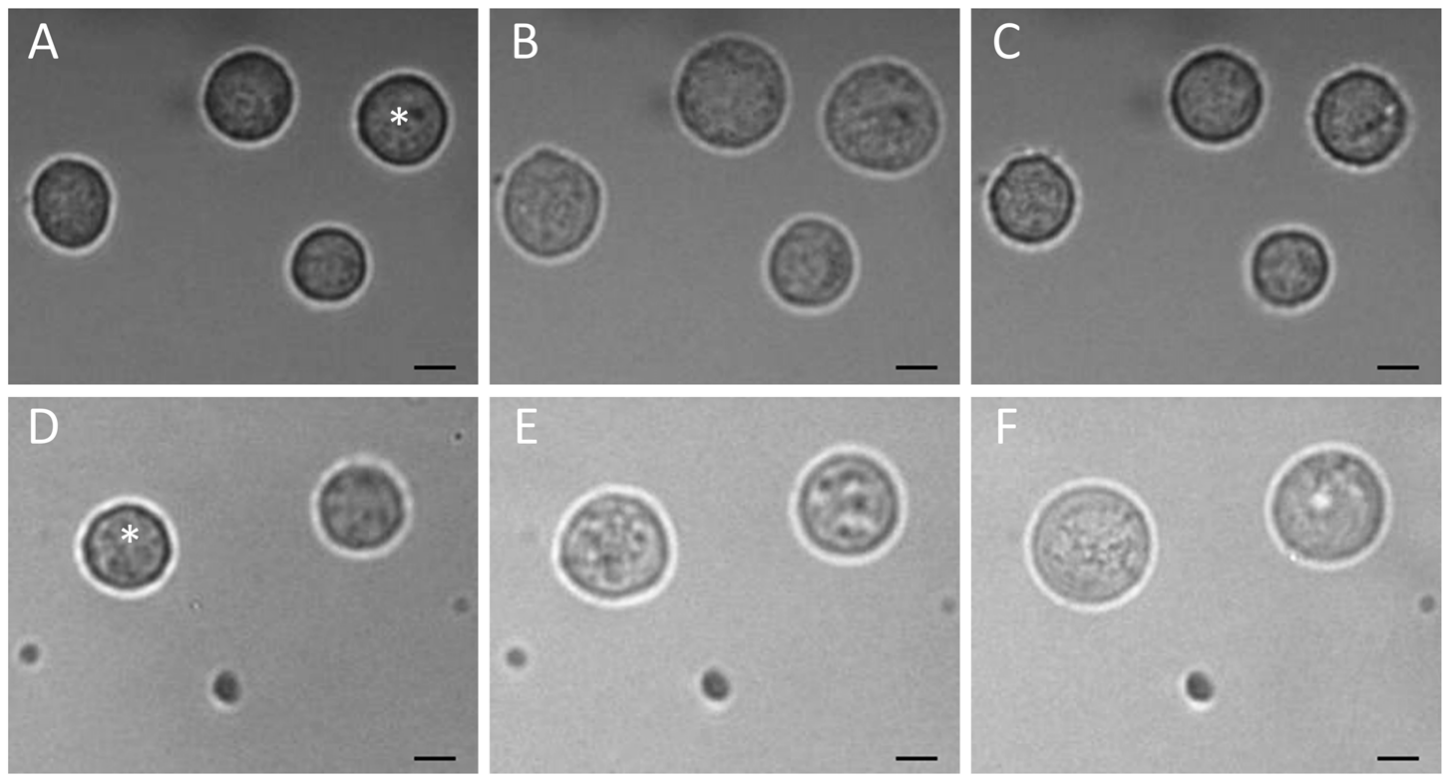
Volume changes in GaMG cells induced by strongly hypotonic sucrose- (*A*–*C*) and inositol-substituted solutions (*D*–*F*) of osmolalities 100 and 50 mOsm, respectively. The microphotographs were taken before (*A* and *D*, isotonic CGM), about 3 min (*B*, *E*) and 20 min (*C*, *F*) after an acute hypotonic shock. In hypotonic sucrose (*A*–*C*), the volume of the indicated cell increased 2.1-fold within 3 min after hypotonic shock (*B*). Thereafter, the cell gradually shrank via the RVD mechanism and reduced its volume to ∼120% of the original isotonic value (*C*). In sharp contrast to the disaccharide sucrose, the small organic osmolyte inositol fully abolished RVD in GaMG cells (*D*–*F*). Thus, upon exposure to 50-mOsm inositol, the cell volume increased first rapidly 1.6-fold at 3 min (*E*), and then slower, more than 3-fold within the following 20 min (*F*). The scale bars correspond to 10 µm.

To compare in detail the osmotic properties of GBM lines, we analyzed the kinetics of cell volume changes during hypotonic stress. To this end, the cells were rapidly transferred from isotonic CGM (∼300 mOsm) to solutions of different osmolalities (300, 100 and 50 mOsm) containing sucrose or inositol as the major solute. As seen in the microphotographs (Figures 4A–4C), 100-mOsm sucrose caused a biphasic volume response of GaMG cells. The cells first swelled rapidly within 2–3 min and then shrank slowly over the following 5–20 min. In contrast, the volume of GaMG cells remained nearly unchanged after the fast initial swelling in 100-mOsm inositol (Figures 4D-4F).

**Figure 5:**
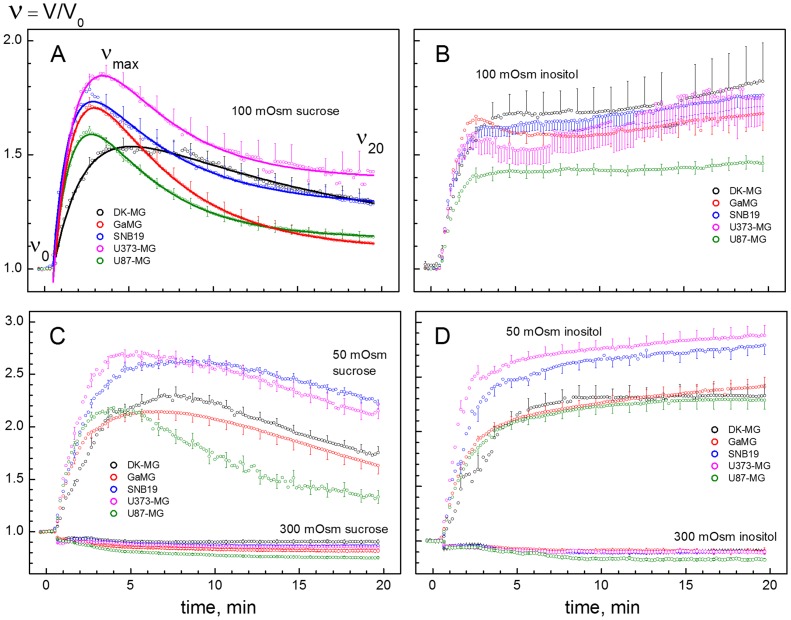
Changes of the normalized volume V/V_0_ in glioblastoma cell lines in response to sucrose- and inositol-substituted solutions of different osmolalities (LHS and RHS columns, respectively). All cells were bathed initially (time <0) in isotonic culture medium (300 mOsm) and then exposed at zero time to solutions having osmolalities of 300, 100 or 50 mOsm. As expected, the initial swelling (2–3 min) of all cell lines increased in magnitude with decreasing osmolality of hypotonic media. But the secondary volume changes (time >5 min) were found to be strongly dependent on both the cell type and the extracellular osmolyte. In general, all tested cell lines were able to undergo regulatory volume decrease (RVD) in sucrose-substituted media over the entire tonicity range (LHS column). In sharp contrast to sucrose, inositol not only abolished RVD but also caused continuous secondary cell swelling, resulting in an up to 3-fold volume increase of GaMG and SNB19 cells 20 min after hypotonic shock (*D*). Each data point represents the mean ± SE of 50–120 individual cells. Continuous curves in *A* show best least-square fits of the Lúcio-model [50] to the data. The fitted parameters (*P*_w_ and α) are given in Table 2. The slower initial swelling of DK-MG cells (*black symbols*) indicates a lower osmotic water permeability of the plasma membrane in this cell line (*P*_w_ in Table 2).

Figure 5 shows the volumetric responses of 5 cell lines to solutions of different osmolalities. The data were derived from microphotographs, such as shown in Figure 4. Independent of the solute used, a sudden exposure to hypotonicity caused all cells to swell rapidly within the first 2–3 min from their original isotonic volume *V*_0_ to the *V*_max_ level due to the fast water influx driven by the imposed osmotic gradient. The rates and magnitudes of initial swelling varied widely among the cell lines. DK-MG cells swelled somewhat slower than other cell lines, which is particularly evident from the data obtained in 50- and 100-mOsm sucrose solutions (black symbols in Figures 5A and 5C). The *V*_max_ values obtained in sucrose media were used to evaluate the osmotically inactive volume fractions β of cells by applying the Boyle van’t Hoff equation (Figure S2). As seen in Table 2, the 5 tested GBM lines exhibited comparable β values, ranging between 0.61 (GaMG) and 0.73 (DK-MG cells).

**Table 2:** Osmotic parameters of glioblastoma cell lines.

Cell line	*a* ± SE µm	β ± SE	*P*_w_ ± SE µm/s	α ± SE nmol/(s⋅cm^2^)	*N*, cell number
**DK-MG**	8.1±0.2	0.73±0.01	0.59±0.10	0.25±0.01	40
**GaMG**	10.0±0.3	0.61±0.05	4.17±0.07	0.46±0.01	30
**U87-MG**	7.6±0.2	0.71±0.02	3.02±0.15	0.37±0.01	50
**U373-MG**	8.8±0.3	0.64±0.08	4.05±0.63	0.35±0.02	20
**SNB19**	8.5±0.4	0.68±0.07	2.21±0.16	0.48±0.02	30

The isotonic cell radius *a* was determined by video microscopy from the cross-sections of cells, such as shown in Figures 4A and 4D. The osmotically inactive volume fraction β was determined from the Boyle van’t Hoff plots (Figure S4). The osmotic water permeability *P*_w_ and the solute permeability during RVD α were determined by fitting the Lúcio-model [50] to the volumetric data obtained with *N* cells (RHS column) in 100-mOsm sucrose solution (Figure 5A).

The data in Figures 4 and 5 reveal a marked difference between sucrose and inositol in their effects on the secondary volume response in all tested cell lines. After the initial swelling in hypotonic sucrose solutions, all GBM lines underwent regulatory volume decrease (RVD). During RVD, the cells shrank gradually despite persisting hypotonicity. RVD relies on the release of cytosolic solutes (including both inorganic ions and small organic osmolytes) through swelling-activated membrane pathways [37], [46]. In agreement with our findings presented here (Figure 5) and previously [47], other glioma cells (including the D54-MG line and primary glioma cells from patient biopsies) are able to readjust their volume in anisotonic media [48].

In sharp contrast to the disaccharide sucrose, the small organic osmolyte inositol not only completely abolished RVD, e.g. in case of DK-MG cells, but also caused noticeable secondary swelling of GaMG and SNB19 cells (Figures 5B and 5D). As shown elsewhere [37], [46], the different cell volume responses to hypotonic inositol and sucrose solutions arise from the size selectivity of swelling-activated membrane pathways, conducting inositol but not sucrose. Mammalian cells ubiquitously express swelling-activated pathways for small organic osmolytes, such as sorbitol, inositol, amino acids etc. [37], [49]. Under our experimental conditions, the influx of extracellular inositol into cells abolished RVD by compensating for the release of intracellular solutes.

Unlike inositol, the disaccharide sucrose did not permeate the plasma membrane of GBM cells, as evidenced by the ability to RVD over the entire hypotonicity range (Figures 5A and 5B). The presence of RVD allowed us to quantitatively analyze the membrane transport properties in terms of the osmotic water and swelling-activated solute permeabilities (*P*_w_ and α, respectively), by applying the Lúcio-model [50]. Best least-square fits of the Lúcio-model to the volumetric data are illustrated by curves in Fig. 5A. The fitted *P*_w_ and α values are given in Table 2.

Among the 5 GBM lines, DK-MG cells exhibited the lowest permeability coefficients for water *P*_w_ and solutes α (Table 2). Based on the optical cell size determination, these quantities, however, neglect the actual cell surface areas associated with folds and microvilli. Consequently, the relatively low *P*_w_ and α values obtained for DK-MG cells (Table 2) can be explained by their smaller membrane area due to a less pronounced membrane folding (φ = 2.38, Table 1), as compared to other tested GBM lines (3.50≤ φ ≤5.25, Table 1).

### Expression of p53, MDM2, PTEN, FAS and Marker Proteins of the PI3K/AKT/mTOR Pathway

**Figure 6:**
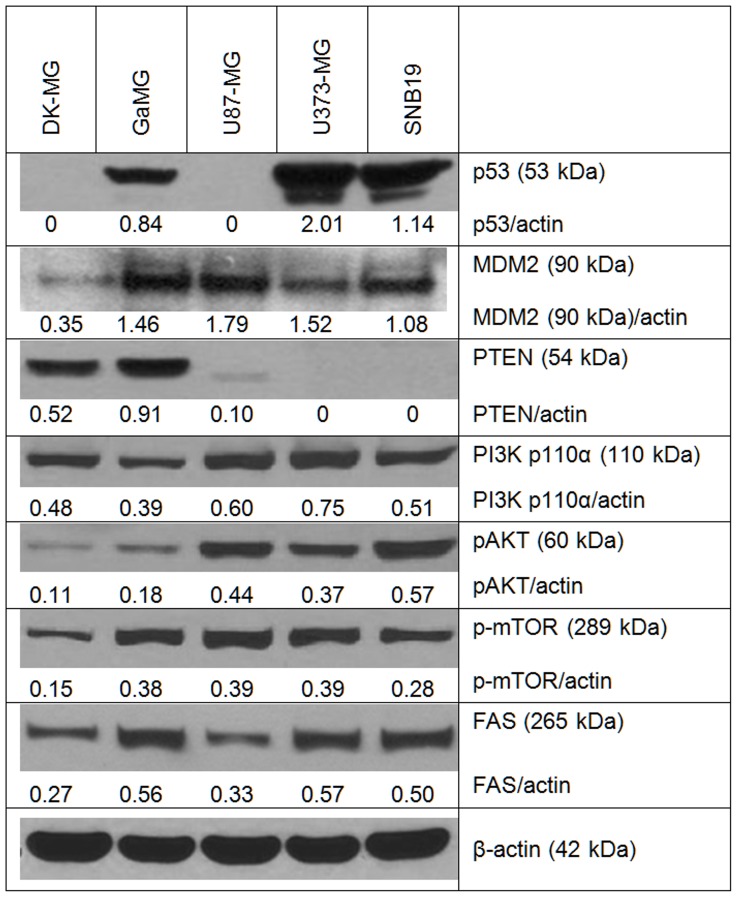
Representative Western blot analysis of the expression of p53, MDM2, PTEN, PI3K, phospho-AKT, phospho-mTOR and FAS proteins. For each cell line, cell lysates were prepared from exponentially growing cells, 20–24 h after splitting the culture. Each protein band was normalized to the intensity of β-actin used as loading control, and the ratios protein/actin are depicted by numbers. The experiments were repeated at least three times.

To elucidate possible molecular mechanisms underlying the observed differences in membrane area and folding among 5 GBM lines, we analyzed by Western blotting the expression of PTEN, p53 and several marker proteins of the PI3K/AKT/mTOR pathway, which is known to be involved in tumor cell growth and invasion. We also determined the levels of FAS (fatty acid synthase), a key enzyme in *de novo* lipogenesis and membrane synthesis. In a previous study [21], elevated levels of FAS protein have been found in various GBM lines and human glioma tissue samples. Figure 6 shows exemplarily the Western blot data of cell samples probed for p53, MDM2, PTEN, PI3K (p110α), phospho-AKT, phospho-mTOR, and FAS.

As seen in Figure 6, the expression of p53 protein varied markedly among the GBM lines. In DK-MG and U87-MG cells, p53 expression was very poor or under the limit of the detection, which is typical for wild type p53 glioblastoma cells [51]. On the other hand, high p53 protein levels 2.0, 1.14 and 0.84 a.u. were found, respectively, in U373-MG, SNB19 and GaMG cell lines, containing mutated p53 gene. The results on p53 expression obtained here are best explained by the fact that wt p53 protein normally has a very short half-life because of its rapid proteasomal degradation [52]. Degradation of wt p53 is regulated by a feedback control of its trans-activating function, involving induction of MDM2, which in turn targets p53 for degradation. When mutant p53 loses its trans-activating function, it cannot induce MDM2 and therefore is not degraded, being thus apparently overexpressed [52]. This mechanism can be responsible for the high p53 expression found in the GBM lines mutated in this gene (Figure 6).

As expected, PTEN protein was detected only in DK-MG and GaMG cells which are wild type PTEN [53]. On the other hand, PTEN mutated U373-MG and SNB19 cell lines showed no expression of PTEN at all (Figure 6), whereas the PTEN mutated U87-MG cells showed very poor expression of PTEN. In agreement with the literature [54], [55], the PI3K/AKT/mTOR pathway was activated in PTEN-mutated cell lines (Figure 6). The expression levels of PI3K and phospho-AKT in PTEN-mutated cells were much higher than in DK-MG and GaMG cells, which can be associated with the lack of PTEN in these cells leading to a compensatory activation of the PI3K pathway. The expression of phospho-mTOR, which regulates protein and lipid synthesis, was up-regulated in most cell lines except DK-MG.

We also analyzed the protein levels of fatty acid synthase (FAS). Among 5 GBM lines, DK-MG cells expressed the lowest levels of FAS protein (0.27 a.u.; Figure 6, bottom line). The highest FAS expression was found in GaMG and U373-MG cells (0.56–0.57 a.u.). Our finding that U87-MG and SNB19 cells expressed comparable levels of FAS protein (0.33–0.50 a.u.) corroborates well the results of Zhao et al. (2006), obtained for the same cell lines [21].

## Discussion

Although derived from the same tumor entity, the 5 GBM lines studied here were considerably different not only in their morphological appearance but also in the density of membrane folds and microvilli, as evident from the SEM images shown in Figure 1. Particularly, DK-MG cells exhibited a fairly smooth apical membrane with irregular blebs and microvilli (Figure 1A-1B), as contrasted to the heavily villated surface of U373-MG and SNB19 cells (Figure 1G–1K).

Our ROT measurements of the area-specific capacitance *C*_m_ also confirm the large variability in membrane folding among 5 GBM cell lines. Thus, in agreement with the relatively smooth appearance of DK-MG cells in SEM images (Figure 1), the membrane capacitance per unit area *C*_m_ [µF/cm^2^] of this cell line was much lower than in other tested GBM cells (Table 1). The *C*_m_ data yield the following ascending rank order of membrane folding for 5 GBM lines: DK-MG (*C*_m_ = 1.88 µF/cm^2^)<<U87-MG (2.82) ≈ GaMG (3.17)<<SNB19 (3.71) ≈ U373-MG (4.00).

Numerous studies have shown that *C*_m_ is highly characteristic for a particular cell type or line. Thus, human erythrocytes usually exhibit a *C*_m_ of 0.7–0.9 µF/cm^2^, typical for smooth membranes [19], [56]–[58]. In case of more complex membrane morphologies, such as in Jurkat T lymphocytes, different experimental methods, including the patch clamp, dielectrophoresis and ROT techniques, have yielded much higher, but very close *C*_m_ values of 1.35–1.40 µF/cm^2^ [37], [59], [60]. The consistency of cell type-specific *C*_m_ values determined by independent experimental approaches suggests this dielectric quantity as a reliable biophysical marker of a particular cellular phenotype.

In addition to *C*_m_ data, our ROT measurements provided the values of the whole-cell capacitance *C*_C_ (pF) for each GBM line. Unlike the area-specific *C*_m_, which reflects membrane folding and microvilli expression, *C*_C_ takes account of the entire electrically accessible membrane area. As with *C*_m_, we found the *C*_C_ values to vary greatly among GBM lines, but the rank order of cell lines with respect to *C*_C_ was completely different: DK-MG (9.4 pF)<U87-MG (15.9)<SNB19 (21.6)<U373-MG (25.3)<GaMG (28.8) (Table 1). Interestingly, the DK-MG cell line, which is the only cell line with wild type PTEN and wild type p53, exhibited the lowest *C*_m_ and the lowest *C*_C_ values among tested GBM lines. As also evident from Table 1, the high *C*_C_ value of GaMG cells (rank 5) was mainly due to their larger radius (8.5 µm), as compared to DK-MG (6.3 µm). In contrast, the large *C*_C_ of U373-MG cells (rank 4) was dominated by the extreme membrane folding (*C*_m_ = 4.0 µF/cm^2^, rank 5), rather than by the moderate size (radius = 7.2 µm).

Qualitatively, the *C*_C_ values measured here by ROT compare favorably with the few published *C*_C_ data of GBM cells obtained in electrophysiological studies. Thus, the mean *C*_C_ = 15.9 pF of U87-MG cells reported here (Table 1) is within the range of 14–40 pF measured for this cell line by the patch-clamp technique (*see* Figure 1H in [61]). On the other hand, the *C*_C_ of 110±10 pF detected in patch-clamped U373-MG cells [62] is almost four times that measured here by ROT for the same cell line (i.e. 28.5±0.7 pF, Table 1). Considering that a relatively small number of U373-MG cells (N = 17 cells) was analyzed by the patch-clamp method [62], the reported *C*_C_ may have been biased in favor of the easily patchable oversized cells.

In the present study, the adherently growing GBM cells were detached by trypsin/EDTA treatment prior to ROT measurements. It can therefore be argued that the *C*_C_ and *C*_m_ values of suspended cells would not properly reflect the original membrane properties of the anchorage-dependent GBM cells. Recently, however, numerous adherent tumor cell lines have been found to conserve their plasma membrane area and cell volume after being enzymatically released into suspension [19]. Accordingly, the *C*_m_ and *C*_C_ values measured on suspended GBM cells reflect the original membrane structure in native adherent state.

A deeper inspection of the dielectric data along with the mutational status of cell lines (Table 1) reveals a striking relationship between membrane folding, probed by *C*_m_, and the presence of *PTEN/p53* mutations. As already mentioned, DK-MG cells bearing wild type *PTEN* and wild type *p53* showed the lowest *C*_m_ values of 1.88 µF/cm^2^ (rank 1 in the ascending order). The two lines possessing one mutation in either *p53* (GaMG) or *PTEN* (U87-MG), exhibited much higher *C*_m_ values, respectively, of 3.2 and 2.8 µF/cm^2^ (ranks 3 and 2). Interestingly, the cell lines with the highest *C*_m_ values (ranks 5 and 4), *i.e.* U373-MG (4.0 µF/cm^2^) and SNB19 (3.7 µF/cm^2^) are mutated in both *PTEN* and *p53*. Being twice as high as the *C*_m_ of DK-MG cells, the very high *C*_m_ values of U373-MG and SNB19 cells corroborate well with the strongly villated membranes of these cell lines revealed by SEM in the present study (Figure 1) and elsewhere (*see* Figure 2A in [13]). The above findings suggest a possible impact of *PTEN* and *p53* mutations on the extent of membrane folding in GBM cells.

**Figure 7:**
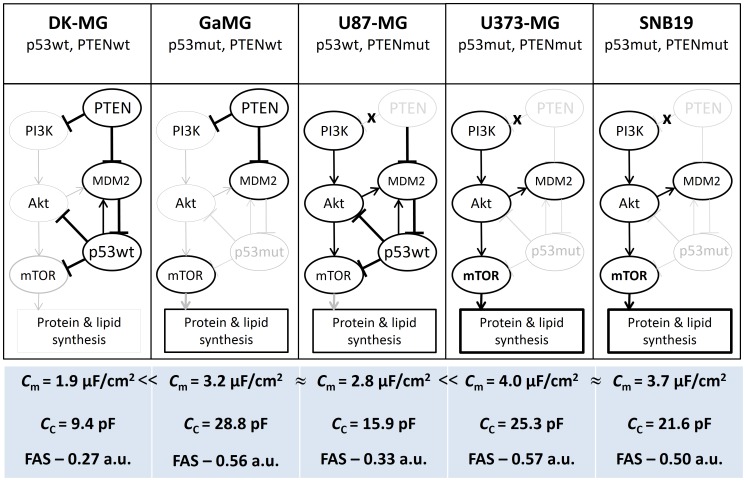
Simplified diagram of putative signaling pathways accountable for the excessive cell membrane area/folding in 5 GBM cell lines differing in their *PTEN* and *p53* mutation. The cell lines are arranged in ascending order according to the degree of membrane folding, probed by the area-specific membrane capacitance *C*_m_. Resulting from the deficiency of either PTEN (U87-MG), p53 (GaMG), or both proteins (U373-MG and SNB19), the overexpression of mTOR (Figure 6) promotes synthesis of proteins and lipids which can be used for the plasma membrane production. The amplified membrane synthesis may lead to the excessive membrane area and folding as reflected, respectively, by a very large *C*_C_ value of GaMG cells and increased *C*_m_ data of U373-MG and SNB19 cells. In addition to deregulation of the PI3K/AKT/mTOR and p53 pathways, the large surface areas of GaMG and U373-MG cells (*C*_C_ = 28.8 and 25.3 pF) may be associated with the increased levels of fatty acid synthase (FAS, 0.56–0.57 a.u.), a key enzyme of the lipogenic pathway.

The observed differences in membrane folding/area among tested GBM lines can be explained by a simplified model illustrated in Figure 7. The model takes into account the expression of marker proteins belonging to the PI3K/AKT/mTOR pathway, as well as of p53, PTEN and FAS, analyzed by Western blotting (Figure 6). Among the tested proteins, mTOR (mammalian target of rapamycin) is known as the major regulator of cell growth and metabolism, whereas FAS (fatty acid synthase) is the key enzyme in the *de novo* lipogenesis (and thus membrane synthesis). The model in Figure 7 also allows for the fact that PTEN and p53 proteins are functionally related to each other, acting as indirect inhibitors of mTOR [55]. Dysregulation of the mTOR signaling pathway is known to be implicated in various types of cancer including GBM [63]. On the cellular level, active mTORC1 (mTOR Complex 1) promotes cell growth and proliferation not only by triggering protein and lipid synthesis but also by reducing autophagy and lysosome biogenesis [55]. In accordance with the inhibitory activity of PTEN and p53 on mTOR, an increased expression of mTOR was found in GaMG, U87-MG, U373-MG and SNB19 cells, *i.e.* those containing mutations in either p53 or PTEN, or both tumor suppressors (Figure 6). As seen in Figure 6, cell lines deficient for PTEN and/or p53 exhibited a more than 3-fold elevated level of phospho-mTOR, as compared to DK-MG cells (wt PTEN and wt p53).

The p53 protein antagonizes mTOR activation by enhancing the activity of AMPK, which inhibits mTORC1 directly or through the activation of TSC1/2 [55]. Interestingly, phospho-mTOR levels in GaMG cells (mut p53, wt PTEN) were similar to the corresponding data of U87-MG (mut PTEN, wt p53) and U373-MG cells (mut PTEN, mut p53), as seen in Figure 6.

In addition to the above mechanisms of mTOR regulation, there is a body of evidence that PTEN and p53 can mutually enhance each other’s expression and activity [30], [64]. The lack of this positive feedback loop between PTEN and p53 pathways can therefore be responsible for the increased levels of phospho-mTOR in GaMG (mut p53) and U87-MG lines (mut PTEN), over those detected in DK-MG cells with wt PTEN and wt p53 (Figure 6).

Besides the mTOR activation mechanism discussed above, the large variations in membrane area among GBM lines (*C*_C_ varies between 9.4 and 28.8 pF, Table 1) may also be associated with the different expression levels of FAS (fatty acid synthase), revealed by immunoblotting. As seen in Figure 6, the levels of FAS protein in GaMG and U373-MG cells (0.56–0.57 a.u.) were more than twice as high as that in DK-MG cells (0.27 a.u.). Since FAS catalyzes the *de novo* lipogenesis and membrane synthesis, the elevated expression/activity of this enzyme can also be responsible for the much larger surface areas of GaMG and U373-MG cells (*C*_C_ = 25.3 and 28.8 pF, respectively), as compared to DK-MG cells (*C*_C_ = 9.4 pF).

Comparison of the dielectric membrane properties presented here with the data on the toxicity of various anti-cancer drugs reported in our previous studies [65]–[67] also suggests a possible relationship between membrane folding/area and chemoresistance of GBM lines. Thus, Stingl et al. (2010) have shown that GaMG cell line (*C*_m_ = 3.2 µF/cm^2^, Table 1) is more sensitive to the Hsp90 inhibitor NVP-AUY922, as compared to SNB19 cells (*C*_m_ = 3.7 µF/cm^2^) [65]. Likewise, DK-MG cells, *i.e.* the cell line exhibiting the lowest *C*_m_ (1.9 µF/cm^2^) in the present study (Table 1), have been found to be much more sensitive to the dual PI3K- and mTOR-inhibitor NVP-BEZ235 than U373-MG and SNB19 lines [66]. Interestingly, the two most chemoresistant GBM lines (U373-MG and SNB19) [66] exhibited in the present study the highest degree of membrane folding, as evidenced by their large *C*_m_ values of 4.0 and 3.7 µF/cm^2^ given in Table 1. Moreover, Niewidok et al. (2012) have shown that the lung carcinoma cell line A549 possessing wt p53 and wt PTEN was much more sensitive to the Hsp90 inhibitor NVP-AUY922 [67] than was the SNB19 cell line which is mutated in both p53 and PTEN.

In addition to chemoresistance, changes in tumor suppressor genes (and related plasma membrane properties studied here) may also be involved in the regulation of invasive behavior of GBM cells. Thus, we found recently that two GBM lines U373-MG and SNB19 (mutated in both PTEN and p53), exhibited much higher invasion rates through Matrigel-coated filters (Boyden chamber assay), as compared to DK-MG, GaMG and U87-MG cells (Djuzenova et al., unpublished data). In agreement with this finding, upregulation of PTEN inhibits migration and wound healing properties of glioma cells [68].

Finally, although the data presented here suggest an apparent correlation between morphological features and PTEN/p53 mutational status as well as lipid metabolism, this study does not definitely prove a causal relationship between genetic features and cellular behavior. Therefore, further studies are warranted to determine if manipulation of PTEN/p53 status and/or lipid metabolism can indeed be directly related to changes in cellular morphology, invasiveness and chemoresistance. Further studies on animal models are also necessary to determine the relationship of these morphological features to behavior of malignant glioma cells *in vivo*.

### Concluding Remarks

The comparative analysis of the dielectric plasma membrane properties revealed a striking difference among 5 established GBM cell lines, most notably, in the degree of membrane folding probed by the area-specific capacitance *C*_m_. Moreover, *C*_m_ was found to correlate with the mutational status of the tumor suppressor genes *PTEN* and *p53*. In accordance with dielectric data, SEM showed various membrane extensions in GBM cells, including folds, microvilli and lamellipodia. Although this study involved only established GBM lines, the ROT technique presented here is easily applicable to any malignant cells including those derived from primary tumors. Given that membrane extensions may be implicated in tumor cell motility and metastasis, the dielectric approach presented here provides a rapid and simple tool for screening tumor cell properties in studies on targeting chemo- or radiotherapeutically the migration and invasion of GBM and other tumor cells.

## Supporting Information

Figure S1 **The radius-normalized** ***f*** **_c1_ values (** ***f*** **_c1_⋅a) of the indicated GBM lines plotted** ***vs.*** **the external conductivity σ_e_.** The measurements were performed in strongly hypotonic 50-mOsm inositol medium. The lines are best fits of Eq. 2 to the data. The fitted *C*_m_ values are summarized in Table S2. For detail *see* text and the Legend to Fig. 3.
(TIF)

Figure S2 **The mean** ***C*** **_m_ (± SE) values of 5 GBM lines compared by the Student’s** ***t*** -test, using the Software Origin 8 (Microcal, Northampton, MA): (*) denotes ***P*** **<0.05;** ***n.s.*** **indicates that the difference was not significant (** ***P*** **>0.05).** The differences in *C*_m_ between GBM lines were statistically significant, except for the pair U373-MG *vs*. SNB19 cells, i.e. the two cell lines mutated in both PTEN and p53.
(TIF)

Figure S3 **Determination of the area-specific membrane capacitance of two non-cancerous, adherently growing cell lines, including the human embryonic kidney HEK293 cells and the human fibroblast cells HFIB-1.** The measurements were performed in isotonic 300-mOsm inositol medium. The *f*_c1_ data were obtained by the CRF-technique. Each symbol is the mean (±SE) from 16–20 cells measured at closely similar conductivities. The lines are best fits of Eq. 2 to the CRF data sets, containing ∼300 HEK293 cells and ∼400 HFIB-1 cells. The fitted *C*_m_ values are 1.56±0.10 and 2.05±0.12 µF/cm^2^ respectively, for HEK293 and HFIB-1 cell lines.
(TIF)

Figure S4
**Boyle van’t Hoff plots for the indicated GBM cell lines.** Each data point represents the mean ν_max_ value (±SE, as defined in Fig. 5A) plotted against the reciprocal normalized osmolality (*conc*_iso_/*conc*, where *conc*_iso_ = 300 mOsm). The data were fitted by the Boyle van’t Hoff equation: 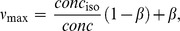 where *conc* is the solution osmolality, the isotonic osmolality is *conc*_iso_ = 300 mOsm, the term β represents the osmotically inactive volume fraction at 300 mOsm. From the Y-intercepts, the β values were found for each cells and summarized in Table 2.(TIF)

Table S1 **Best-fit parameters of the Lorentzian function (Eq. 1) to the ROT spectra of 5 GBM cell lines, such as shown in** **Fig. 2** **.**
(DOCX)

Table S2 **Cell radius and dielectric properties of GBM cells in strongly hypotonic medium of osmolality 50 mOsm.** The *C*_m_ and *C*_C_ values were derived from the data shown in Fig. S1.
(DOCX)

## References

[1] Knobbe CB, Reifenberger G (2003) Genetic alterations and aberrant expression of genes related to the phosphatidyl-inositol-3′-kinase/protein kinase B (Akt) signal transduction pathway in glioblastomas. brain pathology 13: 507–518. 14655756 10.1111/j.1750-3639.2003.tb00481.x PMC8095764

[2] Boström J, Cobbers JM, Wolter M, Tabatabai G, Weber RG, et al (1998) Mutation of the PTEN (MMAC1) tumor suppressor gene in a subset of glioblastomas but not in meningiomas with loss of chromosome arm 10q. Cancer Res 58: 29–33. 9426052

[3] Kraus JA, Glesmann N, Beck M, Krex D, Klockgether T, et al (2000) Molecular analysis of the PTEN, TP53 and CDKN2A tumor suppressor genes in long-term survivors of glioblastoma multiforme. J Neurooncol 48: 89–94. 11083071 10.1023/a:1006402614838

[4] Stupp R, Mason WP, van den Bent MJ, Weller M, Fisher B, et al (2005) Radiotherapy plus concomitant and adjuvant temozolomide for glioblastoma. N Engl J Med 352: 987–996. 15758009 10.1056/NEJMoa043330

[5] Hofer S, Herrmann R (2001) Chemotherapy for malignant brain tumors of astrocytic and oligodendroglial lineage. J Cancer Res Clin Oncol 127: 91–95. 11216919 10.1007/s004320000171 PMC12164992

[6] Yamanaka R, Saya H (2009) Molecularly targeted therapies for glioma. Ann Neurol 66: 717–729. 20035507 10.1002/ana.21793

[7] Giese A, Bjerkvig R, Berens ME, Westphal M (2003) Cost of migration: invasion of malignant gliomas and implications for treatment. J Clin Oncol 21: 1624–1636. 12697889 10.1200/JCO.2003.05.063

[8] Reardon DA, Nabors LB, Stupp R, Mikkelsen T (2008) Cilengitide: an integrin-targeting arginine-glycine-aspartic acid peptide with promising activity for glioblastoma multiforme. Expert Opin Investig Drugs 17: 1225–1235. 10.1517/13543784.17.8.1225 PMC2832832 18616418

[9] Drappatz J, Norden AD, Wen PY (2009) Therapeutic strategies for inhibiting invasion in glioblastoma. Expert Rev Neurother 9: 519–534. 19344303 10.1586/ern.09.10

[10] Black PM, Kornblith PL, Davison PF, Liszczak TM, Merk LP, et al (1982) Immunological, biochemical, ultrastructural, and electrophysiological characteristics of a human glioblastoma-derived cell culture line. J Neurosurg 56: 62–72. 6275048 10.3171/jns.1982.56.1.0062

[11] Bilzer T, Stavrou D, Dahme E, Keiditsch E, Bürrig KF, et al (1991) Morphological, immunocytochemical and growth characteristics of three human glioblastomas established in vitro. Virchows Arch A Pathol Anat Histopathol 418: 281–293. 1708926 10.1007/BF01600156

[12] Machado CML, Zorzeto TQ, Bianco JER, Rosa RG, Genari SC, et al (2009) Ultrastructural characterization of the new NG97ht human-derived glioma cell line using two different electron microscopy technical procedures. Microsc Res Tech 72: 310–316. 19009596 10.1002/jemt.20653

[13] Hess MW, Pfaller K, Ebner HL, Beer B, Hekl D, et al (2010) Chapter 27-3D Versus 2D Cell culture: Implications for electron microscopy. In: Müller-Reichert T, editor. Methods in cell biology. Academic Press, Vol. 96: 649–670. 10.1016/S0091-679X(10)96027-5 20869542

[14] Friedl P, Wolf K (2003) Tumour-cell invasion and migration: diversity and escape mechanisms. Nat Rev Cancer 3: 362–374. 12724734 10.1038/nrc1075

[15] Zaguia F, Schneider R (2011) Microvilli expressed on glioma cells keep cytotoxic cells at a distance. Cancer Biol Ther 11: 1–3. 21189451 10.4161/cbt.11.1.14498

[16] Hoa N, Ge L, Kuznetsov Y, McPherson A, Cornforth AN, et al (2010) Glioma cells display complex cell surface topographies that resist the actions of cytolytic effector lymphocytes. J Immunol 185: 4793–4803. 20855883 10.4049/jimmunol.1001526

[17] Teodori L, Albertini MC, Uguccioni F, Falcieri E, Rocchi MBL, et al (2006) Static magnetic fields affect cell size, shape, orientation, and membrane surface of human glioblastoma cells, as demonstrated by electron, optic, and atomic force microscopy. Cytometry A 69: 75–85. 16419064 10.1002/cyto.a.20208

[18] Ohira K, Homma KJ, Hirai H, Nakamura S, Hayashi M (2006) TrkB-T1 regulates the RhoA signaling and actin cytoskeleton in glioma cells. Biochem Biophys Res Commun 342: 867–874. 16500620 10.1016/j.bbrc.2006.02.033

[19] Gascoyne PRC, Shim S, Noshari J, Becker FF, Stemke-Hale K (2013) Correlations between the dielectric properties and exterior morphology of cells revealed by dielectrophoretic field-flow fractionation. Electrophoresis 34: 1042–1050. 23172680 10.1002/elps.201200496 PMC3754903

[20] Han S-I, Joo Y-D, Han K-H (2013) An electrorotation technique for measuring the dielectric properties of cells with simultaneous use of negative quadrupolar dielectrophoresis and electrorotation. Analyst 138: 1529–1537. 23353873 10.1039/c3an36261b

[21] Zhao W, Kridel S, Thorburn A, Kooshki M, Little J, et al (2006) Fatty acid synthase: a novel target for antiglioma therapy. Br J Cancer 95: 869–878. 16969344 10.1038/sj.bjc.6603350 PMC2360524

[22] Menendez JA, Lupu R (2007) Fatty acid synthase and the lipogenic phenotype in cancer pathogenesis. Nat Rev Cancer 7: 763–777. 17882277 10.1038/nrc2222

[23] Scott KEN, Wheeler FB, Davis AL, Thomas MJ, Ntambi JM, et al (2012) Metabolic regulation of invadopodia and invasion by acetyl-CoA carboxylase 1 and de novo lipogenesis. PLoS ONE 7: e29761 10.1371/journal.pone.0029761 22238651 PMC3253107

[24] Bigner DD, Bigner SH, Pontén J, Westermark B, Mahaley MS, et al (1981) Heterogeneity of genotypic and phenotypic characteristics of fifteen permanent cell lines derived from human gliomas. J Neuropathol Exp Neurol 40: 201–229. 6260907 10.1097/00005072-198105000-00001

[25] Giannini C, Scheithauer BW (1997) Classification and grading of low-grade astrocytic tumors in children. Brain Pathol 7: 785–798. 9161729 10.1111/j.1750-3639.1997.tb01064.x PMC8098338

[26] Akimoto J, Namatame H, Haraoka J, Kudo M (2005) Epithelioid glioblastoma: a case report. Brain Tumor Pathol 22: 21–27. 18095100 10.1007/s10014-005-0173-6

[27] Ohgaki H, Kleihues P (2007) Genetic pathways to primary and secondary glioblastoma. Am J Pathol 170: 1445–1453. 17456751 10.2353/ajpath.2007.070011 PMC1854940

[28] Smith JS, Tachibana I, Passe SM, Huntley BK, Borell TJ, et al (2001) PTEN mutation, EGFR amplification, and outcome in patients with anaplastic astrocytoma and glioblastoma multiforme. JNCI J Natl Cancer Inst 93: 1246–1256. 11504770 10.1093/jnci/93.16.1246

[29] Duncan CG, Killela PJ, Payne CA, Lampson B, Chen WC, et al (2010) Integrated genomic analyses identify ERRFI1 and TACC3 as glioblastoma-targeted genes. Oncotarget 1: 265–277. 21113414 10.18632/oncotarget.137 PMC2992381

[30] Li Y, Guessous F, Kwon S, Kumar M, Ibidapo O, et al (2008) PTEN has tumor-promoting properties in the setting of gain-of-function p53 mutations. Cancer Res 68: 1723–1731. 18339852 10.1158/0008-5472.CAN-07-1963 PMC3813002

[31] Chow LML, Baker SJ (2006) PTEN function in normal and neoplastic growth. Cancer Letters 241: 184–196. 16412571 10.1016/j.canlet.2005.11.042

[32] Rutka JT, Giblin JR, Dougherty DY, Liu HC, McCulloch JR, et al (1987) Establishment and characterization of five cell lines derived from human malignant gliomas. Acta Neuropathol 75: 92–103. 2829496 10.1007/BF00686798

[33] Kennedy PG, Watkins BA, Thomas DG, Noble MD (1987) Antigenic expression by cells derived from human gliomas does not correlate with morphological classification. Neuropathol Appl Neurobiol 13: 327–347. 3317103 10.1111/j.1365-2990.1987.tb00190.x

[34] Westphal M, Haensel M, Mueller D, Laas R, Kunzmann R, et al (1988) Biological and karyotypic characterization of a new cell line derived from human gliosarcoma. Cancer Res 48: 731–740. 3275500

[35] Weller M, Felsberg J, Hartmann C, Berger H, Steinbach JP, et al (2009) Molecular predictors of progression-free and overall survival in patients with newly diagnosed glioblastoma: a prospective translational study of the German Glioma Network. J Clin Oncol 27: 5743–5750. 19805672 10.1200/JCO.2009.23.0805

[36] Sukhorukov V, Djuzenova C, Arnold W, Zimmermann U (1994) DNA, protein, and plasma-membrane incorporation by arrested mammalian cells. J Membr Biol 142: 77–92. 7707355 10.1007/BF00233385

[37] Kiesel M, Reuss R, Endter J, Zimmermann D, Zimmermann H, et al (2006) Swelling-activated pathways in human T-lymphocytes studied by cell volumetry and electrorotation. Biophys J 90: 4720–4729. 16565059 10.1529/biophysj.105.078725 PMC1471856

[38] Gascoyne PRC (2009) Dielectrophoretic-field flow fractionation analysis of dielectric, density, and deformability characteristics of cells and particles. Anal Chem 81: 8878–8885. 19791772 10.1021/ac901470z PMC3754901

[39] Gassert E, Avota E, Harms H, Krohne G, Gulbins E, et al (2009) Induction of membrane ceramides: a novel strategy to interfere with T lymphocyte cytoskeletal reorganisation in viral immunosuppression. PLoS Pathog 5: e1000623 10.1371/journal.ppat.1000623 19834551 PMC2757718

[40] Arnold WM, Zimmermann U (1988) Electro-rotation: development of a technique for dielectric measurements on individual cells and particles. J Electrostat 21: 151–191.

[41] Jones TB (2005) Electromechanics of Particles. Cambridge University Press. 289 p.

[42] Lei U, Sun P-H, Pethig R (2011) Refinement of the theory for extracting cell dielectric properties from dielectrophoresis and electrorotation experiments. Biomicrofluidics 5: 44109–4410916 10.1063/1.3659282 22662061 PMC3364808

[43] Asami K (2011) Dielectric properties of microvillous cells simulated by the three-dimensional finite-element method. Bioelectrochem 81: 28–33. 10.1016/j.bioelechem.2011.01.002 21333613

[44] Schroeder TE (1979) Surface area change at fertilization: resorption of the mosaic membrane. Dev Biol 70: 306–326. 478164 10.1016/0012-1606(79)90030-7

[45] Lange K (2011) Fundamental role of microvilli in the main functions of differentiated cells: Outline of an universal regulating and signaling system at the cell periphery. J Cell Physiol 226: 896–927. 20607764 10.1002/jcp.22302

[46] Sukhorukov VL, Imes D, Woellhaf MW, Andronic J, Kiesel M, et al (2009) Pore size of swelling-activated channels for organic osmolytes in Jurkat lymphocytes, probed by differential polymer exclusion. Biochim Biophys Acta 1788: 1841–1850. 19560440 10.1016/j.bbamem.2009.06.016

[47] Djuzenova CS, Krasnyanska J, Kiesel M, Stingl L, Zimmermann U, et al (2009) Intracellular delivery of 2-deoxy-D-glucose into tumor cells by long-term cultivation and through swelling-activated pathways: implications for radiation treatment. Mol Med Rep 2: 633–640. 21475878 10.3892/mmr_00000149

[48] Ernest NJ, Weaver AK, Van Duyn LB, Sontheimer HW (2005) Relative contribution of chloride channels and transporters to regulatory volume decrease in human glioma cells. Am J Physiol, Cell Physiol 288: C1451–1460. 15659714 10.1152/ajpcell.00503.2004 PMC2548409

[49] Shennan DB (2008) Swelling-induced taurine transport: relationship with chloride channels, anion-exchangers and other swelling-activated transport pathways. Cell Physiol Biochem 21: 15–28. 18209468 10.1159/000113743

[50] Lúcio A, Santos R, Mesquita O (2003) Measurements and modeling of water transport and osmoregulation in a single kidney cell using optical tweezers and videomicroscopy. Physical Review E 68. doi: 10.1103/PhysRevE.68.041906. 10.1103/PhysRevE.68.041906 14682972

[51] Quick QA, Gewirtz DA (2006) An accelerated senescence response to radiation in wild-type p53 glioblastoma multiforme cells. J Neurosurg 105: 111–118. 16871885 10.3171/jns.2006.105.1.111

[52] Blagosklonny MV (2000) p53 from complexity to simplicity: mutant p53 stabilization, gain-of-function, and dominant-negative effect. FASEB J 14: 1901–1907. 11023974 10.1096/fj.99-1078rev

[53] Michaud K, Solomon DA, Oermann E, Kim J-S, Zhong W-Z, et al (2010) Pharmacologic inhibition of cyclin-dependent kinases 4 and 6 arrests the growth of glioblastoma multiforme intracranial xenografts. Cancer Res 70: 3228–3238. 20354191 10.1158/0008-5472.CAN-09-4559 PMC2855904

[54] Mise-Omata S, Obata Y, Iwase S, Mise N, Doi TS (2005) Transient strong reduction of PTEN expression by specific RNAi induces loss of adhesion of the cells. Biochem Biophys Res Commun 328: 1034–1042. 15707982 10.1016/j.bbrc.2005.01.066

[55] Laplante M, Sabatini DM (2012) mTOR signaling in growth control and disease. Cell 149: 274–293. 22500797 10.1016/j.cell.2012.03.017 PMC3331679

[56] Gimsa J, Müller T, Schnelle T, Fuhr G (1996) Dielectric spectroscopy of single human erythrocytes at physiological ionic strength: dispersion of the cytoplasm. Biophys J 71: 495–506. 8804632 10.1016/S0006-3495(96)79251-2 PMC1233500

[57] Chan KL, Gascoyne PR, Becker FF, Pethig R (1997) Electrorotation of liposomes: verification of dielectric multi-shell model for cells. Biochim Biophys Acta 1349: 182–196. 9421190 10.1016/s0005-2760(97)00092-1 PMC2726258

[58] Asami K (2013) Dielectric properties of dipicrylamine-doped erythrocytes, cultured cells and lipid vesicles. Bioelectrochemistry 92: 14–21. 23523956 10.1016/j.bioelechem.2013.02.003

[59] Ross PE, Garber SS, Cahalan MD (1994) Membrane chloride conductance and capacitance in Jurkat T lymphocytes during osmotic swelling. Biophys J 66: 169–178. 8130336 10.1016/S0006-3495(94)80754-4 PMC1275677

[60] Pethig R, Bressler V, Carswell-Crumpton C, Chen Y, Foster-Haje L, et al (2002) Dielectrophoretic studies of the activation of human T lymphocytes using a newly developed cell profiling system. Electrophoresis 23: 2057–2063. 12210259 10.1002/1522-2683(200207)23:13<2057::AID-ELPS2057>3.0.CO;2-X

[61] Catacuzzeno L, Aiello F, Fioretti B, Sforna L, Castigli E, et al (2011) Serum-activated K and Cl currents underlay U87-MG glioblastoma cell migration. J Cell Physiol 226: 1926–1933. 21506123 10.1002/jcp.22523

[62] Bordey A, Sontheimer H, Trouslard J (2000) Muscarinic activation of BK channels induces membrane oscillations in glioma cells and leads to inhibition of cell migration. J Membr Biol 176: 31–40. 10882426 10.1007/s00232001073

[63] Bliesath J, Huser N, Omori M, Bunag D, Proffitt C, et al (2012) Combined inhibition of EGFR and CK2 augments the attenuation of PI3K-Akt-mTOR signaling and the killing of cancer cells. Cancer Lett 322: 113–118. 22387988 10.1016/j.canlet.2012.02.032

[64] Stambolic V, MacPherson D, Sas D, Lin Y, Snow B, et al (2001) Regulation of PTEN transcription by p53. Mol Cell 8: 317–325. 11545734 10.1016/s1097-2765(01)00323-9

[65] Stingl L, Stühmer T, Chatterjee M, Jensen MR, Flentje M, et al (2010) Novel HSP90 inhibitors, NVP-AUY922 and NVP-BEP800, radiosensitise tumour cells through cell-cycle impairment, increased DNA damage and repair protraction. Br J Cancer 102: 1578–1591. 20502461 10.1038/sj.bjc.6605683 PMC2883148

[66] Kuger S, Graus D, Brendtke R, Günther N, Katzer A, et al (2013) Radiosensitization of glioblastoma cell lines by the dual PI3K and mTOR inhibitor NVP-BEZ235 depends on drug-irradiation schedule. Transl Oncol 6: 169–179. 23544169 10.1593/tlo.12364 PMC3610553

[67] Niewidok N, Wack L-J, Schiessl S, Stingl L, Katzer A, et al (2012) Hsp90 inhibitors NVP-AUY922 and NVP-BEP800 may exert a significant radiosensitization on tumor cells along with a cell type-specific cytotoxicity. Transl Oncol 5: 356–369. 23066444 10.1593/tlo.12211 PMC3470116

[68] Dasari VR, Kaur K, Velpula KK, Gujrati M, Fassett D, et al (2010) Upregulation of PTEN in glioma cells by cord blood mesenchymal stem cells inhibits migration via downregulation of the PI3K/Akt pathway. PLoS ONE 5: e10350 10.1371/journal.pone.0010350 20436671 PMC2859936

