# Supplementary figures and images for: Cell Surface Area and Membrane Folding in Glioblastoma Cell Lines Differing in PTEN and p53 Status
Source: PLoS One. 2014 Jan 31;9(1):e87052. doi: 10.1371/journal.pone.0087052 (PMC3909012; doi:10.1371/journal.pone.0087052)

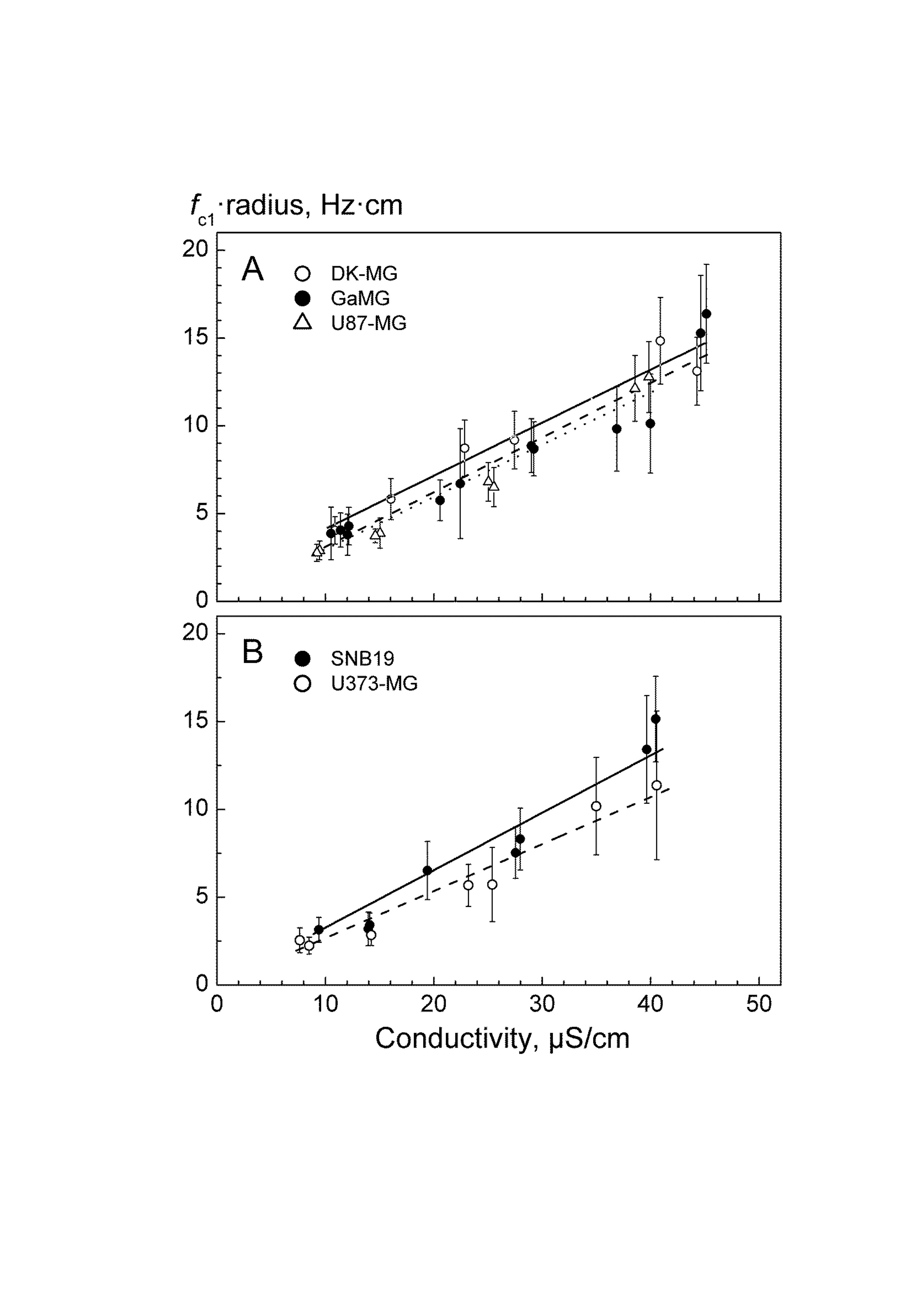

Supplement: Figure S1 — The radius-normalized f c1 values ( f c1⋅a) of the indicated GBM lines plotted vs. the external conductivity σe. The measurements were performed in strongly hypotonic 50-mOsm inositol medium. The lines are best fits of Eq. 2 to the data. The fitted C m values are summarized in Table S2. For detail see text and the Legend to Fig. 3. (TIF) [file pone.0087052.s001.tif]

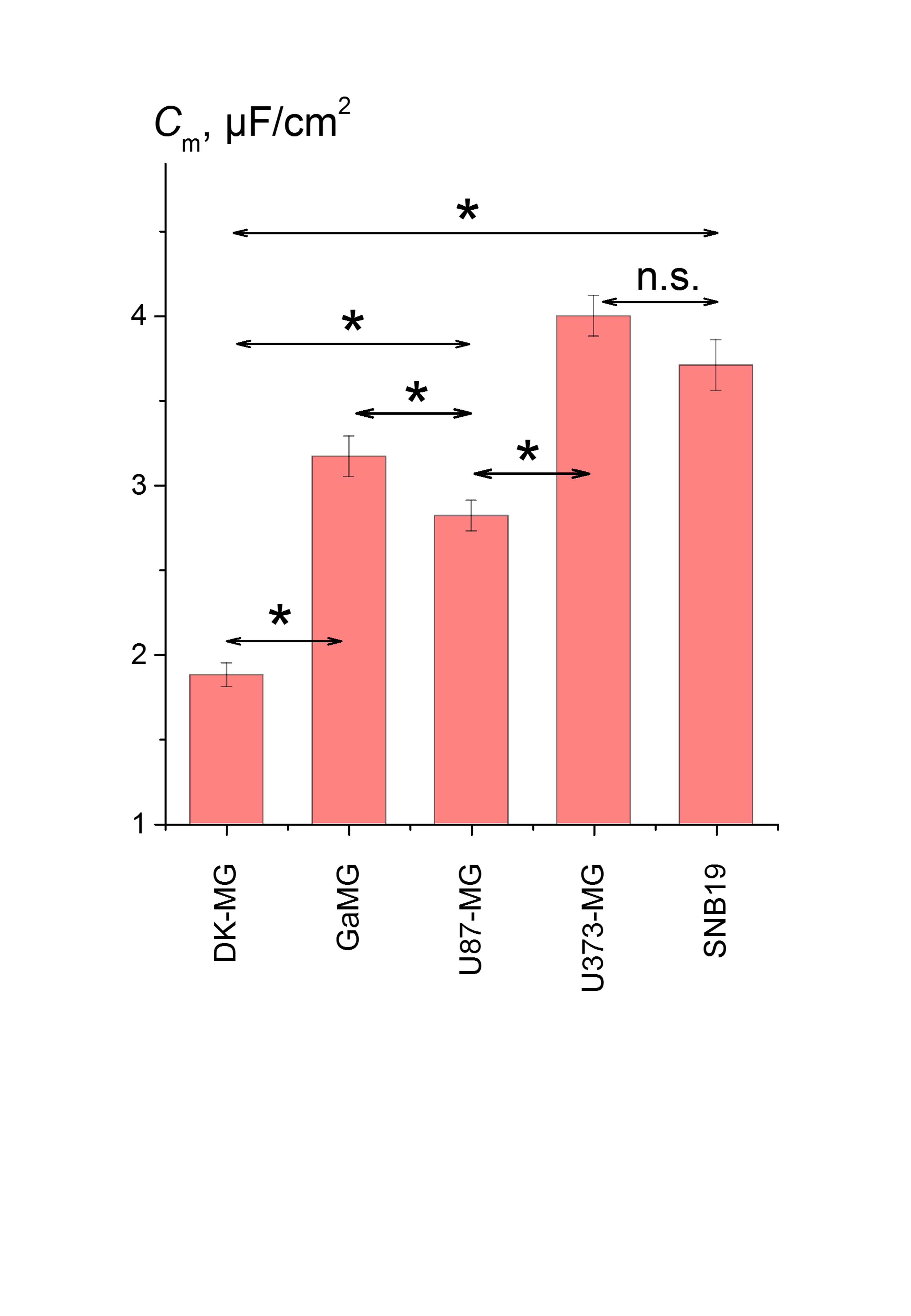

Supplement: Figure S2 — The mean C m (± SE) values of 5 GBM lines compared by the Student’s t -test, using the Software Origin 8 (Microcal, Northampton, MA): (*) denotes P <0.05; n.s. indicates that the difference was not significant ( P >0.05). The differences in C m between GBM lines were statistically significant, except for the pair U373-MG vs. SNB19 cells, i.e. the two cell lines mutated in both PTEN and p53. (TIF) [file pone.0087052.s002.tif]

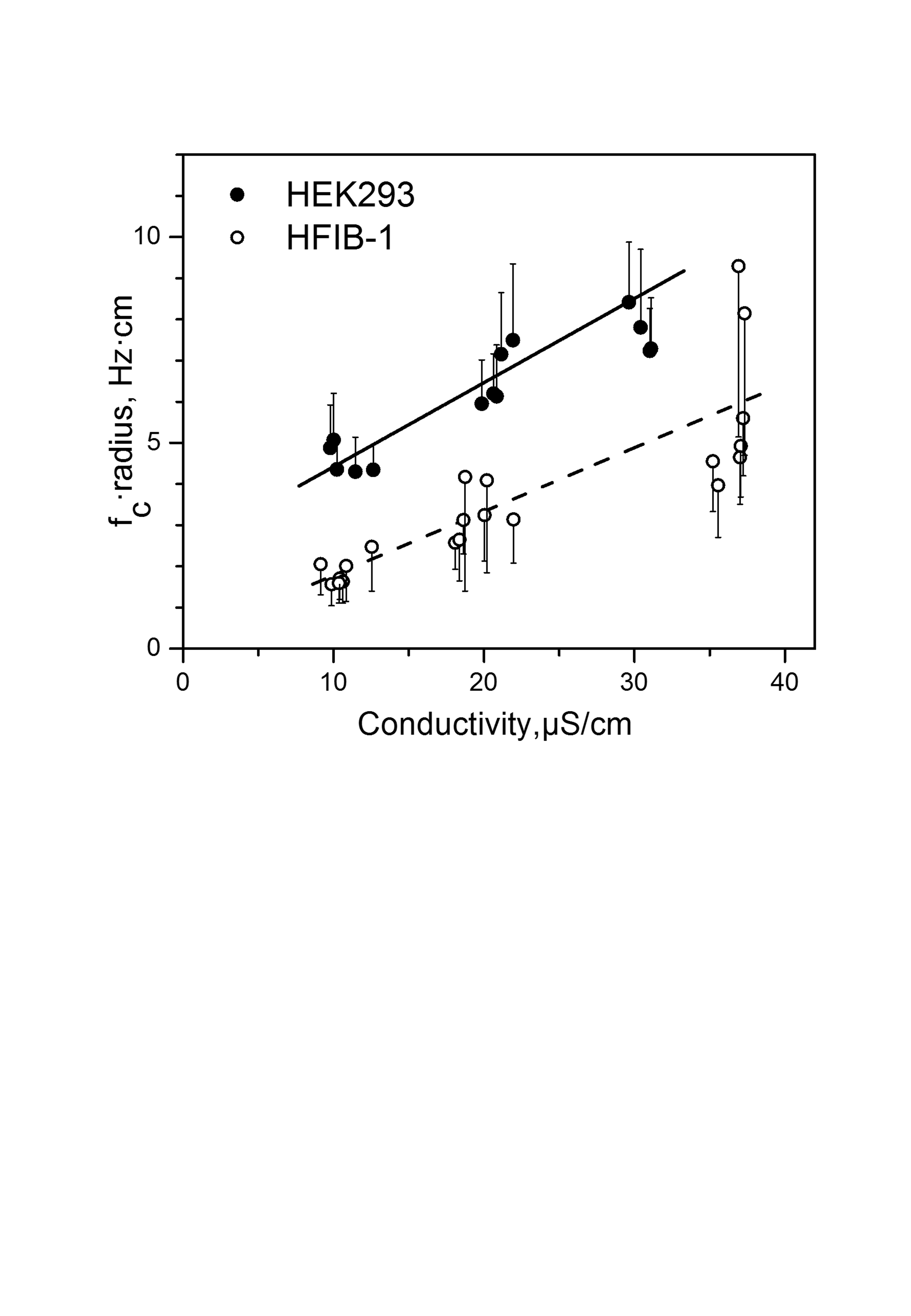

Supplement: Figure S3 — Determination of the area-specific membrane capacitance of two non-cancerous, adherently growing cell lines, including the human embryonic kidney HEK293 cells and the human fibroblast cells HFIB-1. The measurements were performed in isotonic 300-mOsm inositol medium. The f c1 data were obtained by the CRF-technique. Each symbol is the mean (±SE) from 16–20 cells measured at closely similar conductivities. The lines are best fits of Eq. 2 to the CRF data sets, containing ∼300 HEK293 cells and ∼400 HFIB-1 cells. The fitted C m values are 1.56±0.10 and 2.05±0.12 µF/cm2 respectively, for HEK293 and HFIB-1 cell lines. (TIF) [file pone.0087052.s003.tif]

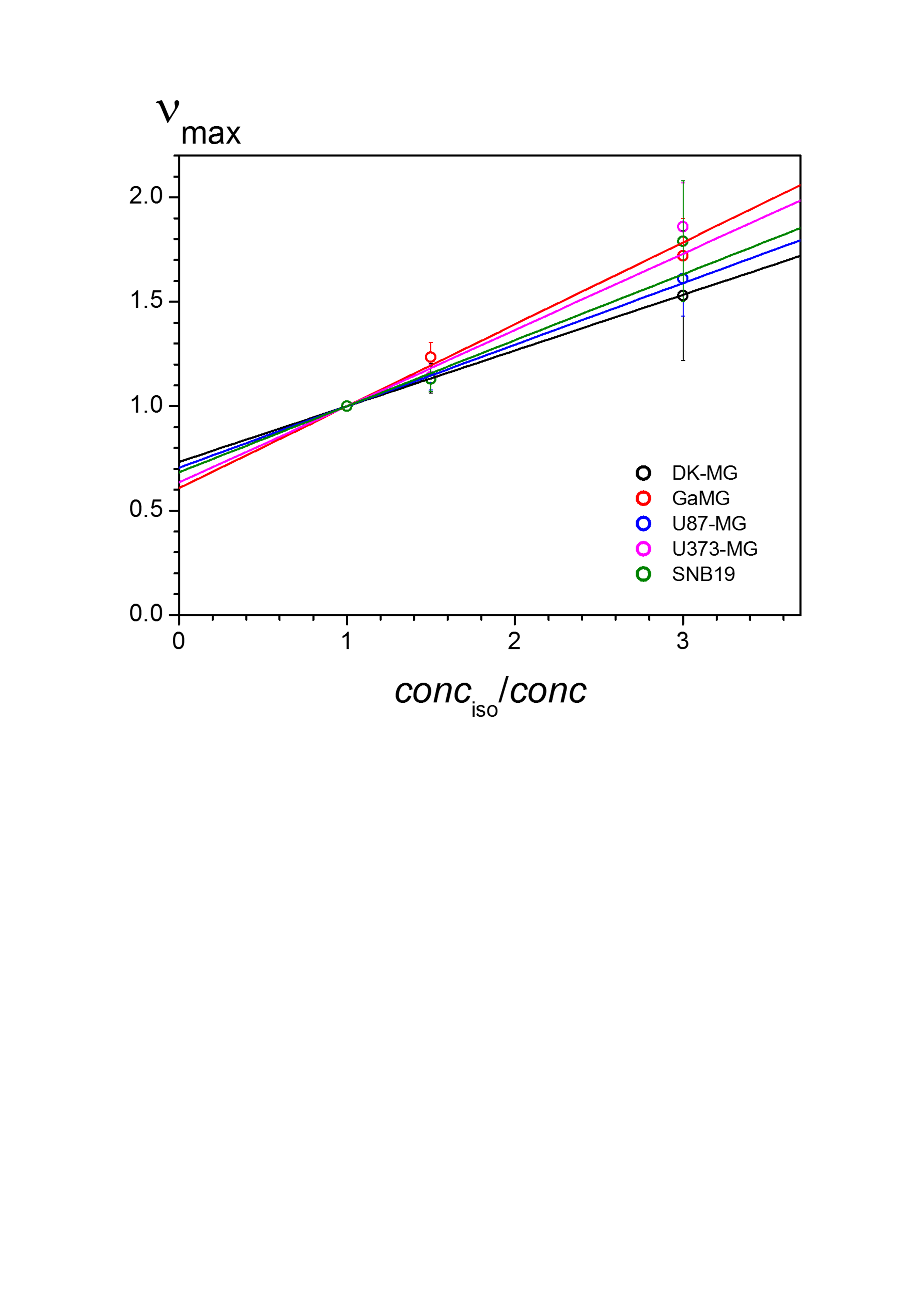

Supplement: Figure S4 — Boyle van’t Hoff plots for the indicated GBM cell lines. Each data point represents the mean νmax value (±SE, as defined in Fig. 5A) plotted against the reciprocal normalized osmolality (conc iso/conc, where conc iso = 300 mOsm). The data were fitted by the Boyle van’t Hoff equation: where conc is the solution osmolality, the isotonic osmolality is conc iso = 300 mOsm, the term β represents the osmotically inactive volume fraction at 300 mOsm. From the Y-intercepts, the β values were found for each cells and summarized in Table 2. (TIF) [file pone.0087052.s004.tif]
