# Supplementary material for: Cell Surface Area and Membrane Folding in Glioblastoma Cell Lines Differing in PTEN and p53 Status
Source: PLoS One. 2014 Jan 31;9(1):e87052. doi: 10.1371/journal.pone.0087052 (PMC3909012; doi:10.1371/journal.pone.0087052)
Supplement: Table S1 — Best-fit parameters of the Lorentzian function (Eq. 1) to the ROT spectra of 5 GBM cell lines, such as shown in Fig. 2 . (DOCX) [file pone.0087052.s005.docx]

**Table S1. Best-fit parameters of the Lorentzian function (Eq. 1) to the ROT spectra of 5 GBM cell lines^*^**

| **Cell line** | **Log_10_(*f*_c1_) [*f*_c1_, Hz]** | ***A*_1_ [radian/sec]** | **Log_10_(*f*_c2_) [*f*_c2_, Hz]** | ***A*_2_ [radian/sec]** | **Log_10_(*f*_c3_) [*f*_c3_, Hz]** | ***A*_3_ [radian/sec]** | **r^2^** | **N** |
| --- | --- | --- | --- | --- | --- | --- | --- | --- |
| **DK-MG** | 4.22±0.01 | -3.15±0.07 | 6.67±0.05 | 0.97±0.09 | 7.63±0.07 | 1.10±0.10 | 0.993 | 10 |
| **GaMG** | 3.87±0.04 | -2.70±0.19 | 6.65±0.13 | 0.55±0.14 | 7.63±0.12 | 1.26±0.17 | 0.966 | 10 |
| **U87-MG** | 4.12±0.01 | -2.16±0.05 | 6.72±0.07 | 0.77±0.12 | 7.55±0.08 | 0.88±0.12 | 0.992 | 6 |
| **U373-MG** | 3.84±0.03 | -1.70±0.08 | 6.67±0.05 | 0.56±0.05 | 7.63±0.05 | 0.72±0.04 | 0.993 | 10 |
| **SNB19** | 3.79±0.02 | -2.20±0.05 | 6.52±0.03 | 1.07±0.04 | 7.68±0.04 | 1.10±0.05 | 0.997 | 9 |

* All spectra were measured at a conductivity of about 50 µS/cm. The data represent the means ± SE from *N* individual cells.
