# Supplementary material for: Cell Surface Area and Membrane Folding in Glioblastoma Cell Lines Differing in PTEN and p53 Status
Source: PLoS One. 2014 Jan 31;9(1):e87052. doi: 10.1371/journal.pone.0087052 (PMC3909012; doi:10.1371/journal.pone.0087052)
Supplement: Table S2 — Cell radius and dielectric properties of GBM cells in strongly hypotonic medium of osmolality 50 mOsm. The C m and C C values were derived from the data shown in Fig. S1. (DOCX) [file pone.0087052.s006.docx]

**Table S2: Cell radius and dielectric properties of GBM cells in strongly hypotonic medium of osmolality 50 mOsm**

| **Cell line** | **Radius ± SE µm** | ***C*_mh_** **± SE µF/cm^2^** | ***C*_Ch_** **± SE pF** | **N, cell number** |
| --- | --- | --- | --- | --- |
| **DK-MG** | 10.2 ± 0.1 | 1.05 ± 0.05 | 13.7 ± 0.3 | 120 |
| **GaMG** | 13.3 ± 0.1 | 1.02 ± 0.02 | 22.7 ± 0.4 | 240 |
| **U87-MG** | 10.4 ± 0.1 | 1.13 ± 0.02 | 15.3 ± 0.2 | 221 |
| **U373-MG** | 12.2 ± 0.1 | 1.19 ± 0.03 | 22.1 ± 0.4 | 140 |
| **SNB19** | 11.6 ± 0.1 | 0.97 ± 0.02 | 16.4 ± 0.3 | 160 |
